# Current Strategies and Applications for Precision Drug Design

**DOI:** 10.3389/fphar.2018.00787

**Published:** 2018-07-18

**Authors:** Chen Wang, Pan Xu, Luyu Zhang, Jing Huang, Kongkai Zhu, Cheng Luo

**Affiliations:** ^1^School of Biological Science and Technology, University of Jinan, Jinan, China; ^2^Drug Discovery and Design Center, State Key Laboratory of Drug Research, Shanghai Institute of Materia Medica, Chinese Academy of Sciences, Shanghai, China; ^3^School of Pharmacy, University of Chinese Academy of Sciences, Beijing, China; ^4^School of Pharmacy, Fudan University, Shanghai, China

**Keywords:** precision medicine, precision drug design, computational modeling, deep learning, antibody-drug conjugates, ligand-targeted conjugates

## Abstract

Since Human Genome Project (HGP) revealed the heterogeneity of individuals, precision medicine that proposes the customized healthcare has become an intractable and hot research. Meanwhile, as the Precision Medicine Initiative launched, precision drug design which aims at maximizing therapeutic effects while minimizing undesired side effects for an individual patient has entered a new stage. One of the key strategies of precision drug design is target based drug design. Once a key pathogenic target is identified, rational drug design which constitutes the major part of precision drug design can be performed. Examples of rational drug design on novel druggable targets and protein–protein interaction surfaces are summarized in this review. Besides, various kinds of computational modeling and simulation approaches increasingly benefit for the drug discovery progress. Molecular dynamic simulation, drug target prediction and *in silico* clinical trials are discussed. Moreover, due to the powerful ability in handling high-dimensional data and complex system, deep learning has efficiently promoted the applications of artificial intelligence in drug discovery and design. In this review, deep learning methods that tailor to precision drug design are carefully discussed. When a drug molecule is discovered, the development of specific targeted drug delivery system becomes another key aspect of precision drug design. Therefore, state-of-the-art techniques of drug delivery system including antibody-drug conjugates (ADCs), and ligand-targeted conjugates are also included in this review.

## Introduction

In January 2015, President Obama announced the new Precision Medicine Initiative^1^ to bring human closer to cure diseases like cancer and diabetes and to give all of us the ability to access to the personalized information to keep ourselves and our families in good health. As this initiative launched, precision drug design has entered a new stage. Cell-based HTS method has been widely used to obtain drug candidates, however, the hits identified by this method are usually difficult to undergo further optimization because of their bad lipophilic characteristic (Congreve et al., 2011). Since the first protein structure was determined by X-ray crystallography in the 1960s, medicinal chemists have made use of the three-dimensional structures of therapeutically relevant targets to guide the design of new therapeutic agents (Thomas et al., 2017). The availability of three-dimensional structures of therapeutically relevant proteins and protein–protein interaction surfaces allows the characterization and identification of potential modulator binding sites and lays the foundation for SBDD. In recent years, SBDD has shed light on precision drug design and accelerated the progress toward the new era of precision medicine. Medicinal chemists and computational chemists have made great progress in the field of SBDD since the early development of inhibitors that target thymidylate synthase and viral neuraminidase for the treatment of cancer and influenza disease in the 1980s (Jaskolski et al., 2014). Besides, in the discovery of the four United States Food and Drug Administration (FDA)-approved antiviral protease inhibitors (saquinavir, nelfinavir, indinavir, and ritonavir) for the treatment of Human immunodeficiency virus infection and acquired immune deficiency syndrome (HIV/AIDS), the contributions of the structure of HIV protease cannot be ignored. With the exponential growth of the therapeutically relevant target structures being deposited in Protein Data Bank (PDB), precision drug design has entered a new era.

Motivated by Precision Medicine Initiative, computational modeling and simulation approaches have been widely used to provide new biomarkers, predict potential intercellular mechanisms, and decipher protein conformational changes and cell behavior. Among them, molecular dynamics simulation is a popular computational technique that could not only help to understand the macromolecular conformational changes and the correspondingly biological function which may indicate novel pathogenic mechanisms but also help to characterize the flexible binding sites and pathways, kinetics, and thermodynamics (Liu et al., 2018; Śledź and Caflisch, 2018). Besides, computational modeling, through analyzing and integrating numerous biological data, has been successfully applied in basic science and drug development which presents unique opportunities for developing novel treatment options (Zhang and Brusic, 2014). In this review, the application of computational modeling in drug target prediction and *in silico* clinical trials, as well as the application of molecular dynamics simulation in ligand identification and optimization will be discussed.

Machine learning has been widely applied to drug discovery and design, from the first use of ANN in classifying molecules as active or inactive in the early 1970s, to the application of ANN for quantitative structure-activity relationship analysis (Baskin et al., 2016). Deep learning based machine learning algorithms are just starting out on the journey to drug discovery and precision drug design, though they have already swept across the areas of image classification, distributed representations and language processing (LeCun et al., 2015). Unlike conventional machine learning algorithms which need specific experts to design good feature extractors for subsequent analysis, deep learning could automatically extract complex patterns among massive data, which may be suitable for genomic data mining and other biological problems (LeCun et al., 2015; Gawehn et al., 2016). Thanks to the advances of different architectures for solving potential deep learning optimization challenges and the enhanced computing power especially GPU, deep learning has benefited many hot fields, such as medical imaging for diagnosis of diseases and precision medicine. Many companies like Atomwise, IBM Watson, and Gritstone have initiated research programs to implement artificial intelligence in drug development and precision medicine (Mesko, 2017).

Antibody-drug conjugates have experienced rapid development in recent years and their homogeneity, solubility, stability, and efficacy have been improved owing to exquisite design and iterative optimization. ADCs are considered as precise weapons that direct against and kill the antigen expressed cells while spare the healthy ones (Drachman and Senter, 2013). The development, characteristics and perspectives of the three generation ADCs are reviewed. Besides, ligand-targeted therapeutic or imaging conjugates are also summarized in this review.

Overall, computational approaches have lowered the barriers and provided unique opportunities in drug development. In this review, current state-of-the-art technologies including target-specific *de novo* drug design, computational modeling and simulations, deep learning, and antibody-drug or ligand-targeted conjugates are detailly discussed.

## Target-Specific *De Novo* Drug Design

Discovery of potent and selective modulators for therapeutically relevant targets has been encouraged by a better understanding of protein-ligand and protein–protein interactions as well as detailed structural information of molecular recognition. In recent years, various strategies and techniques have been developed to facilitate *de novo* design of target-specific modulators.

### Virtual Screening

**FIGURE 1:**
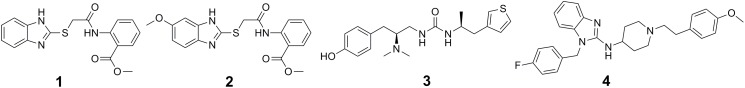
Chemical structures of inhibitors discovered by virtual screening.

The past decade has witnessed rapid development and wide applications of structure-based virtual screening, which has become an attractive alternative to traditional HTS for early stage of drug discovery in both academia and industry. Compared with HTS, it is an effective, low-cost, labor-saving strategy for drug discovery. The use of docking-based virtual screening in the identification of DNA methyltransferases (DNMT1 and DNMT3A) inhibitors has been well reviewed elsewhere (Lu et al., 2018). In another case, to discover novel protein arginine methyltransferase 5 (PRMT5) inhibitors, a pharmacophore and molecular docking based virtual screening was performed followed by bioactivity assay of an initial subset of top-ranked molecules (116 members). Among the six compounds that showed potent PRMT5 inhibitory activity, DC_P04 (1 in **Figure 1**) was selected to undergo further structural optimization. Its derivative 17 (2 in **Figure 1**) was then found to be the most active one, which inhibited PRMT5 enzymatic activity at nanomolar concentration (IC_50_ = 330 nM) and displayed potent anti-proliferative effects on MV4-11 cells (Mao et al., 2017). In 2016, by computational docking over 3 million molecules against the μ-opioid-receptor (μOR) structure, Manglik et al. (2016) identified new scaffolds dissimilar to known opioids. Structure-based optimization yielded PZM21 (3 in **Figure 1**) that served as a therapeutic lead which was devoid of many side effects of current opioids (Manglik et al., 2016). To develop novel inhibitors targeting the protein–protein interaction (PPI) between the polycomb repressive complex 2 (PRC2) catalytic subunit EZH2 and EED, Kong et al. (2014) performed a molecular docking based virtual screening on an in-house database containing approximately 1000 known drugs, and found that the FDA-approved drug astemizole (4 in **Figure 1**), could disrupt the EZH2-EED interaction, destabilize the PRC2 complex and inhibit its methyltransferase activity in cancer cell lines (Kong et al., 2014). This study first proposed the therapeutic strategy for PRC2-driven human cancers via destructing the EZH2-EED complex (Kong et al., 2014). Identification modulators using virtual screening methods targeting other PPI including WDR5-MLL1, and Menin–MLL1 have been well reviewed (Lu et al., 2018).

### Fragment-Based Drug Design

**FIGURE 2:**
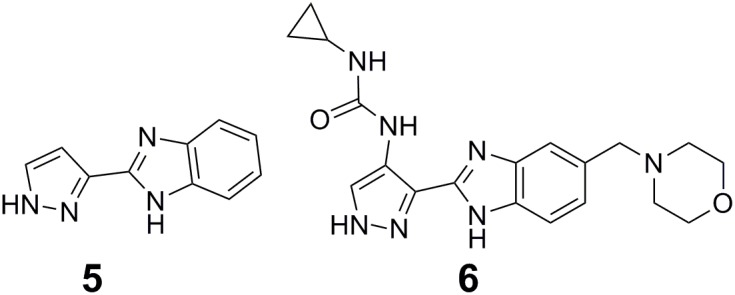
Fragment-based approach for the design of Aurora kinase inhibitors.

Fragment-based drug design has become a powerful approach for precision drug design and been successfully applied to develop potent and selective inhibitors of protein kinases (Erlanson et al., 2004; Howard et al., 2009; Bamborough et al., 2011). The first step of fragment-based drug design is to establish libraries of fragments consisting of low-molecular-weight compounds that have specific interactions and high efficiency with the target protein. The second step is to convert fragments into hits and leads. Fragment optimization, fragment merging or linking, and *in situ* fragment assembly are three broad strategies for converting fragments into hits and leads (Erlanson et al., 2004). Using the pyrazole-benzimidazole 5 (5 in **Figure 2**) as a fragment starting point in the discovery of Aurora kinase inhibitors, Howard et al. performed fragment-based drug design (Howard et al., 2009). The clinical candidate AT9283 (6 in **Figure 2**) for the treatment of cancer targeting on Aurora kinase was finally identified after optimization of the cellular activity and physicochemical properties of the initial fragment (Howard et al., 2009).

### Covalent Targeting of Non-catalytic Cysteine Residues

**FIGURE 3:**
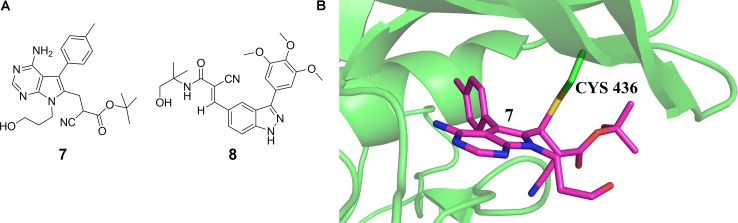
**(A)** Discovery of RSK2-CTD inhibitors via covalent targeting the non-catalytic cysteine residues. **(B)** Cocrystal structure of RSK2-CTD bound to compound 7 (PDB code: 4D9U).

Covalent targeting of non-catalytic cysteine residues is another powerful strategy for improving pharmacological potency and selectivity. Irreversible covalent and reversible covalent binding are two covalent cysteine-targeting strategies. Guided by structural analysis, goal-directed design of the covalent bond formation between an electrophilic ligand and a conserved non-catalytic cysteine of protein target has led to the discovery of selective, irreversible protein kinases (Fry et al., 1998; Cohen et al., 2005; Zhou et al., 2009, 2010; Honigberg et al., 2010) and NS3/4A serine protease (Hagel et al., 2011) inhibitors, though it is difficult to target protein kinases owing to the high sequential and structural conservation of the active pocket. However, due to the concern about unspecific modification of irreversible strategy, the irreversible covalent adducts were currently avoided in drug discovery process. Thus the development of reversible cysteine-targeting strategy was motivated. Serafimova et al. (2012) discovered a derivative of cyanoacrylamide 16 (7 in **Figure 3A**) that inhibited the RSK2 CTD by forming a reversible covalent bond with cysteine 436 (**Figure 3B**) found in only 11 of the 518 human protein kinase (Serafimova et al., 2012). In 2013, based on their previously identified heteroaryl-substituted cyanoacrylamides that were shown to form reversible covalent bonds with cysteine thiols, Miller et al. (2013) identified electrophilic fragments with sufficient ligand efficiency and selectivity to act as starting points for the first reported MSK1 CTD inhibitors. In this case, fragment-based drug design and covalent targeting of non-catalytic cysteine were combined to identify the reversible covalent inhibitor RMM-46 (8 in **Figure 3A**) that exhibited high ligand efficiency and selectivity for MSK/RSK-family kinases (Miller et al., 2013). This compound was shown to block the activation of cellular MSK and RSK activity at nanomolar levels, and inhibit the phosphorylation of the critical transcription factor, CREB (Miller et al., 2013).

### Small Molecule Prodrugs

**FIGURE 4:**
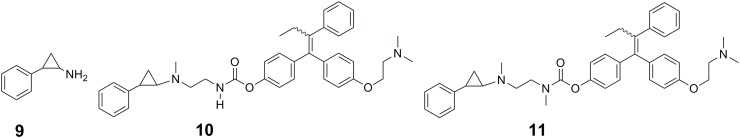
Chemical structures of inhibitors designed by small molecule prodrugs strategy.

Small molecule prodrugs strategy could help overcome problems associated with conventional cancer-targeting methods. Precision design of small molecule prodrugs that can enzymatically or spontaneously spark a chemical reaction at a target site and release a drug to execute an actual drug function has become a novel method in anticancer drug design. As the release of anticancer drugs can be controlled in cancer cells, it can produce good clinical results in cancer therapy. Ota et al. (2016) focused on the FAD-dependent lysine-specific demethylase 1 (LSD1) to trigger the controlled release of anticancer drugs in cancer cells where LSD1 is highly expressed (Ota et al., 2016). LSD1 inhibitor PCPA (9 in **Figure 4**) conjugates were employed as small molecule prodrugs to selectively release anticancer drugs under the mechanism of the PCPA-induced LSD1 inhibition. PCPA-tamoxifen conjugates 1a (10 in **Figure 4**) and 1b (11 in **Figure 4**) that could release 4-hydroxytamoxifen (an anti-estrogen agent for breast cancer treatment) in the presence of LSD1 *in vitro* were designed as PCPA-drug conjugate prototypes. As expected, 1a and 1b exhibited anticancer activity in LSD1 overexpressed breast cancer cells. Further analysis showed that the simultaneous inhibition of LSD1 and the estrogen receptor and the release of 4-hydroxytamoxifen triggered by the inhibition of LSD1 together contributed to the anticancer effectiveness (Ota et al., 2016).

### Mechanism-Based Drug Design

Mechanism-based drug design relies on detailed understanding of the interaction mechanism between the target protein and its ligand, and provides a good basis for precision drug design. This approach is well illustrated by the mechanism-based covalent neuraminidase inhibitors with broad-spectrum influenza antiviral activity and the computational design of a time-dependent histone deacetylase 2 selective inhibitor.

**FIGURE 5:**
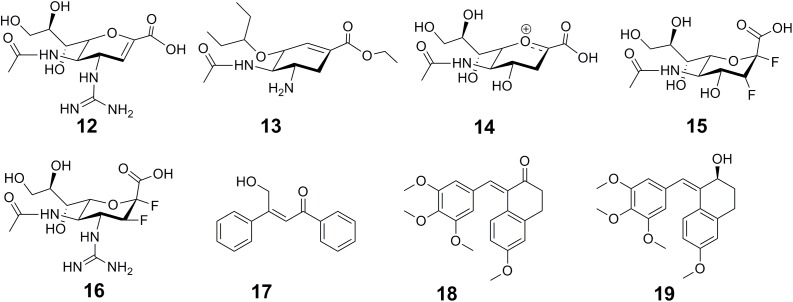
Chemical structures of inhibitors designed through mechanism-based drug design strategy.

The widely used anti-influenza drugs, zanamivir (12 in **Figure 5**) and oseltamivir (13 in **Figure 5**) were designed to mimic the transition state of the natural substrate sialic acid (14 in **Figure 5**), which leaded to the improved activity and specificity on human neuraminidase (NA). To overcome drug resistance, there is an urgent need for the development of novel scaffold of NA inhibitors. Based on the finding that influenza NAs employ a covalent mechanism by using DFSA (15 in **Figure 5**) as a substrate, Kim et al. explored difluorosialic acids (DFSAs) as a possible novel covalent mechanism-based influenza therapeutic agent (Kim et al., 2013). The optimal derivative FeqGuDFSA (16 in **Figure 5**) showed an IC_50_ value comparable to those for zanamivir and oseltamivir. In addition, DFSAs showed good inhibition of NAs for zanamivir or oseltamivir-resistant influenza virus strains (Kim et al., 2013). Most importantly, DFSAs function well in controlling influenza infections in animal model, at levels comparable to those using zanamivir. In another case, the high sequence similarity (97.8%) and the highly conserved residues around the catalytic pocket present difficulties in achieving isoform inhibition selectivity between histone deacetylases HDAC1 and HDAC2. Zhou et al. (2015) developed a *de novo* reaction-mechanism-based inhibitor design strategy guided by their previously characterized HDAC reaction mechanism (Wu et al., 2010), and found a time-dependent HDAC2 selective inhibitor β-hydroxymethyl-chalcone (17 in **Figure 5**) (Zhou et al., 2015). A potent CA-4 like tubulin polymerization inhibitor 22b (18 in **Figure 5**) was found with strong antitumor activity but poor stability. Under natural light, it is inclined to undergo *cis*–*trans* isomerization, resulting in decreased activity. Jiang et al. (2015) elucidated the mechanism of the *cis*–*trans* isomerization by performing quantum chemistry calculation. Inspired by their quantum chemistry calculation results, they designed a series of compounds with improved stability and activity. Among them, compound 1 (19 in **Figure 5**) displayed potent antitumor activity in human colon cancer HT-29 xenograft model (Jiang et al., 2015).

### Bidentate-Binding (Bisubstrate) Inhibitors Design

**FIGURE 6:**
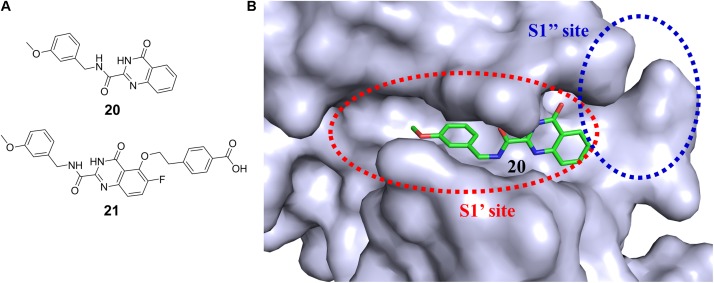
**(A)** Chemical structures of highly potent and selective fused pyrimidine-based MMP-13 inhibitors. **(B)** Surface representation of MMP-13 illustrating the binding cavity. The inhibitor (20) is buried deeply into the S1′ pocket.

It is particularly challenging to discover isoform-selective inhibitors of protein kinase family members, since they share high sequential and structural similarities, especially the conserved ATP binding pocket. Bidentate-binding (bisubstrate) strategy in which a ligand simultaneously occupies the ATP binding site and a unique pocket outside the ATP cleft has been employed to identify selective kinase inhibitors as both tool compounds and potent therapeutic candidates (Asaba et al., 2009; Poot et al., 2009; Lavogina et al., 2010; Lamba and Ghosh, 2012; van Wandelen et al., 2012, 2013; Ekambaram et al., 2013; Rodrik-Outmezguine et al., 2016). The advantage of bisubstrate inhibitors is their ability to generate more interactions with the target protein, which could result in improved affinity and selectivity compared with single-site inhibitors. The availability of this approach will be elucidated by exemplifying its application to the drug discovery of various targets, especially kinase. A moderately active matrix metalloproteinase 13 (MMP-13) inhibitor quinazoline-2-carboxamide (20 in **Figure 6A**) (IC_50_ = 12 nM) was found by HTS (Fabre et al., 2014). The structural basis for the high selectivity of this compound was revealed by the cocrystal complex structure of 20 with MMP-13 (PDB code: 3WV2, Nara et al., 2014a,b). The S1′ pocket of MMPs (**Figure 6B**) has been widely used to modulate ligand selectivity as it displays higher variability in shape and length (Fabre et al., 2014). A series of derivatives of 20 were designed to occupy the distinct deep S1′ pocket and adjacent side pocket. Among them, compound 21 (in **Figure 6A**) showed potent inhibitory activity (IC_50_ = 0.0039 nM) and high selectivity over other MMPs (MMP-1, -2, -3, -7, -8, -9, -10, -14, and TACE) (Nara et al., 2014b). X-ray crystal structure (the PDB code was unreleased) analysis of MMP-13 complexed with 21 confirmed that this compound was indeed buried deep in the S1′ pocket and interacted with an additional S1′ side pocket (S1″ pocket) in MMP-13 (Nara et al., 2014b). Besides, the preliminary repeated-dose oral toxicity study of compound 21 in rats revealed that no overt toxicity was found.

**FIGURE 7:**
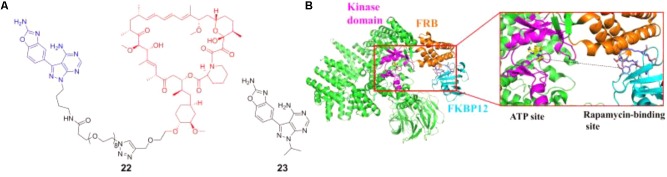
**(A)** Chemical structures of mTOR inhibitor RapaLink-1 and MLN0128. **(B)** Design of bivalent mTOR inhibitor.

Rodrik-Outmezguine et al. (2016) discovered the new-generation mTOR inhibitor RapaLink-1 (22 in **Figure 7A**) that could overcome mTOR resistance mutations by exploiting the unique juxtaposition of two drug-binding pockets (**Figure 7B**) to create a bivalent interaction. In order to simultaneously occupy the ATP binding site and rapamycin-binding site, a bivalent mTOR inhibitor which contained rapamycin, a linker and MLN0128 (23 in **Figure 7A**, an mTOR kinase inhibitor, currently in clinical trials) was designed and synthesized. This compound could potently inhibit tumor growth and mTOR signaling in wild-type mTOR-expressing cells as well as in cells that have acquired resistance to rapalogs or ATP-competitive inhibitors, or both (Rodrik-Outmezguine et al., 2016). This strategy has also been used for the inhibitor discovery of G-protein-coupled receptors, and has been well reviewed elsewhere (Valant et al., 2012).

## Computational Modeling and Simulation Approaches

Given the complexity of biological process and life system, computational approaches that deal with multifarious parameters and features have played essential roles in analyzing systemic response of agents, identifying drug targets, and predicting novel therapeutic strategies (Pennisi et al., 2016). In addition, big data analysis and comprehensive databases have greatly speeded up the drug discovery process (**Table 1**). Here, we summarize computational modeling and simulation approaches that give a helping hand in various drug development process.

**Table 1:** Databases that are useful in basic science and clinical research.

Database	Abbreviation	Website
Gene Expression across Normal and Tumor tissue	GNET	http://medicalgenome.kribb.re.kr/GENT/
The Cancer Genome Atlas	TCGA	https://cancergenome.nih.gov/
Kyoto Encyclopedia of Genes and Genomes	KEGG	http://www.genome.jp/kegg/
Online Mendelian Inheritance in Man	OMIM	http://omim.org/
National Cancer Database	NCDB	https://www.facs.org/quality-programs/cancer/ncdb
Catalogue of Somatic Mutations in Cancer	COSMIC	http://cancer.sanger.ac.uk/cosmic
Immuno Polymorphism Database	IPD	https://www.ebi.ac.uk/ipd/

### Molecular Dynamics (MD) Simulations

Molecular dynamics simulations have been long proposed to unravel novel cryptic binding sites of the targets as well as provide insight into protein dynamics beyond that available crystallographic information. By performing MD simulations on their constructed complex structures of M2 muscarinic acetylcholine receptor (M2 receptor) with its allosteric modulators, Dror et al. (2013) revealed a new binding site that was approximately 15 Å away from the classic recognition site of the M2 receptor, and then validated this novel binding site by radioligand binding experiments. The rational design of allosteric modulators targeting M2 receptor could benefit from these findings. Unbiased simulations of ligand unbinding are useful in provide insight into affinity of the complex (Huang and Caflisch, 2011). As drug efficacy has been proposed to be related with the residence time than that of affinity (Copeland et al., 2006), a lot of simulation protocols have been developed to estimate the kinetic parameters of drug binding/unbinding (Mollica et al., 2015). MD simulations are frequently used to provide insight into hit optimization, especially the hit was obtained from in virtual screening. Zhao et al. (2012) discovered a novel chemotype of inhibitors of the EphB4 tyrosine kinase by fragment-based high-throughput docking followed by explicit solvent MD simulations for assessment of the binding mode. MD simulation results indicated that an additional hydroxyl group involved in two favorable hydrogen bonds may improve the affinity of the hit, thus a single derivative of the hit was synthesized. The enzymatic results showed that this derivative was a true EphB4 inhibitor with nanomolar affinity.

### Drug Target Prediction

Identification of new drug targets for specific cancer types is crucial in drug research pipeline. Big data offers good opportunities to integrate different kinds of data resources and help in data modeling and data correction. In this field, Tao et al. (2015) predicted 15 promising drug targets for colorectal cancer (CRC) through ontology construction and protein–protein interaction network analysis. Among the 15 potential targets, 3 of them have been approved as successful drug targets, indicating that the combination approach of datamining and network analysis could provide promising drug targets for cancer treatment. In another work, Lu et al. (2015) constructed a knowledge-based network revealing the underlying mechanism of colitis-associated colon cancer development. In addition, through Boolean dynamic simulation of the network and *in silico* mutation studies, they identified an important network module and a combinational anti-cancer strategy which targeted ceramide and PI3K-AKT pathway consistently. They further utilize chemical tools to experimentally validate their prediction and successfully observed the synergistic effects in colon cancer cells. Hopefully, the network model could further assist researchers with mechanism studies and drug target prediction. In addition to network analysis, there are many good web servers that can be easily accessed to and be used to predict the drug target and small molecule-protein interactions (**Table 2**). SuperDrug2 provides comprehensive information of approved and marketed drugs and allows similarity and substructure search to obtain target information (Siramshetty et al., 2018). PockDrug server focuses on the pocket druggability using serval pocket estimation models, while PharmMapper identifies drug target using pharmacophore mapping approach (Liu et al., 2010; Hussein et al., 2015).

**Table 2:** Computational online resources used in various field.

Computational	Website
resources	
SuperDRUG2	http://cheminfo.charite.de/superdrug2/drug_search.html
PockDrug	http://pockdrug.rpbs.univ-paris-diderot.fr/cgi-bin/index.py?page=home
PharmMapper	http://lilab.ecust.edu.cn/pharmmapper/index.php
NetMHCpan 4.0 Server	http://www.cbs.dtu.dk/services/NetMHCpan/

### *In Silico* Clinical Trials

Drug research pipeline, especially clinical trial, is a cost-intensive and time-consuming process. In the past few years, the methods of *in silico* clinical trials have been increasingly recommended in drug development in purpose of predicting the safety and efficacy of candidate drugs in an economic manner (Pappalardo et al., 2018; Zhu et al., 2018).

In the field of drug metabolism, glucuronidation by uridine diphosphate-glucuronosyltransferases to the metabolically potential sites of a drug is a critical step for detoxification in living body. Moreover, the pharmacological profile of a drug including systemic exposure and clearance will be affected through this phase II metabolism. Various computational models including classification model, naïve Bayes models and other machine learning methods have been applied in predicting possible sites of glucuronidation. In Peng and coworkers’ work, they built four classification models (aliphatic hydroxyl, carboxylic, aromatic hydroxyl and amino nitrogen) based on support vector machine method and a large chemical dataset (Peng et al., 2014). Model assessment revealed good predicting performance on compound test and they also suggested molecules with higher lipophilicity and small size are more liable to glucuronidation. The prediction among metabolic site and pharmacokinetic properties using *in silico* approaches could accelerate the process of lead optimization and help researchers make rational decisions.

In the field of immunology, differential equation-based models and agent-based model have been utilized in modeling immune system. Differential equation-based approach is a mathematical model that describes organism-wide cell population dynamics in continuous time. Differential equation-based models are usually more effective in grabbing the dominating cellular and molecular change under bacterial or viral attack (Regoes et al., 2007). However, when facing more complicated scenarios, an equation-based model is often hard to construct due to the tradeoff between mathematical feasibility and realistic biology signatures (Pappalardo et al., 2014). In another aspect, Kaufman first introduced automaton model in immunology, and many efforts have been made to optimize this technology (Kaufman et al., 1985). Of note, Celada and Seiden successfully developed their simulator ImmSimm to investigate immune receptor specific behaviors and since then cellular automata and agent-based model have become popular in modeling immune system (Celada and Seiden, 1992; Celada and Seiden Philip, 1996; Figge et al., 2008). In contrast to differential equation-based models, cellular automata and agent-based models are discrete-time stochastic cellular automaton and the global behaviors are integrated by all involved entities (Pappalardo et al., 2014). Furthermore, its flexibility allows researchers to add and delete certain entities and non-linear interaction according to different issues. Recently, Pappalardo et al. (2016) established a relatively comprehensive pipeline framework for virtual screening of citrus-derived vaccine adjuvants in the model of influenza A. Strikingly, their simulator that based on agent-based model carefully considered the crucial cellular interaction occurred in the influenza A affected organism, and good consistency was obtained between *in silico* prediction and *in vivo* validation. The above work is a good attempt that utilizes agent-based models to help discover new drug. Owing to the ideal flexibility and feasibility of agent-based modeling approach in immunology, it is promising to further design patient specific model for the prediction of immunology treatment.

## Deep Learning Based Genetic Variant Discovery and Neoantigen Prediction

**FIGURE 8:**
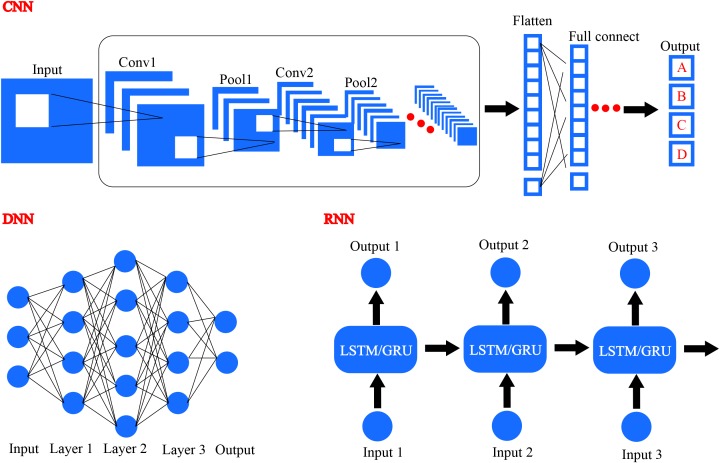
Several different frameworks of deep learning, including convolutional neural network (CNN), deep neural network (DNN), and recurrent neural network (RNN).

With the revival of deep learning based artificial intelligence, different deep learning algorithms have been applied in the area of drug design and precision medicine. Common deep learning algorithms comprise DNN, CNN, and RNN, as illustrated in **Figure 8** (LeCun et al., 2015). Generative RNNs have been used for *de novo* molecular design by Schneider’s group and they utilized RNN with long short term memory (LSTM) cells to construct a SMILES-based compound generation model (Gupta et al., 2017). Matthew and coworkers trained and evaluated a CNN model to score the docking poses generated by docking program smina, and they used Caffe deep learning framework to define the model (Ragoza et al., 2017). In addition, various deep learning methods have been developed to investigate the potential of artificial intelligence in drug discovery and precision medicine design.

### Genetic Variants

It has long been known that one of the most important aspects of precision medicine is to accurately describe the genomes of patients or their tumors from a single individual level, since cancer is recognized as a mosaic genetic disorder (Biesecker and Spinner, 2013). By systematic interrogation of cancer genomes, it will eventually make great contributions to targeted drug design, disease diagnosis and more impressive clinical therapeutic decisions (Boehm and Hahn, 2011). It is estimated that there are 3 billion base pairs of human genome, of which only 1% represents the protein coding genes, however, 85% mutations are existing in the coding genes which could result in diverse diseases (Choi et al., 2009). According to Lauschke and Ingelman-Sundberg (2016), a single genome could harbor 10,000–12,000 altered protein sequences owing to the average 4.1–5 million SNP within each genome. All these statistical data benefits from the giant leap of NGS technologies and the steep decline of sequencing costs, which at the same time demonstrates the heterogeneity and complexity of cancer.

Currently, NGS mainly consists of genome-based high-throughput sequencing platforms including but not limited to WGS and WES, and transcriptome-based high-throughput sequencing like RNA-seq (Carter and He, 2016; Goodwin et al., 2016). RNA sequencing is used to sequence all transcripts within cells to detect the specific expression levels of genes in a given condition such as drug treatment. When compared with normal control, the differentially expressed genes could be analyzed, which is now widely implemented in biological studies (Han et al., 2015). WGS, especially WES, has a great impact on clinical prognosis and therapy, as well as clinical assessment. By incorporating patient’s full genome sequence analysis, researchers could find the single nucleotide polymorphisms and copy number variations in a patient to further predict the genetic risk of diseases like cancer and metabolic diseases (Euan et al., 2010). Moreover, molecular diagnosis based on significant mutation sites within disease-related genes would contribute to clinical practice, which is largely attributable to the advancement of NGS (Choi et al., 2009). Routine framework for genomic data processing comprises several procedures including quality control based data preprocessing, sequencing reads alignment, variant calling and variant annotation (Carter and He, 2016). For reads alignment, many programs such as Burrows-Wheeler alignment tool, Bowtie2 and Novoalign, could align the sequencing reads to the reference genome for subsequent comparison (Li and Durbin, 2009; Langmead and Salzberg, 2012). Variant calling, the key step for detection mutations, has proved challenging due to the complex source of errors not only from sequencing biases introduced by instruments, but also resulting from alignment and calculation algorithms (Li, 2014). Even state-of-the-art variant callers like GATK (McKenna et al., 2010), which use statistical models to identify the possible variants, could only achieve high accuracy to a certain extent (Li, 2014). In 2016, FDA launched the precision FDA Truth Challenge for advancing the quality standards in the genomic community and promoting better personalized care. DeepVariant, developed by Google, outperformed the existing tools in terms of the SNP-Fscore and was awarded the highest SNP performance, which demonstrated the powerful ability of deep learning and its potential applications in precision therapy (Poplin et al., 2017).

DeepVariant is constructed based on the original Inception-v2 architecture, a CNN framework commonly used in image recognition developed by Google (Szegedy et al., 2015). Unlike common image classification, of which the input features are pixel values, DeepVariant utilizes the aligned sequencing reads and reference genome to construct a pseudo image with three channels including aligned bases, corresponding quality scores and read features. After training the CNN model with labeled variants, DeepVariant achieved excellent generalization ability to accurately predict the genotype likelihoods for given sites. Of note, variant callers are largely depended on the sequencing platforms, and manual parameter retuning is necessary when generalizing these statistical models to other sequencing technologies (Goodwin et al., 2016). However, because DeepVariant is only based on deep learning algorithms and has no specialized genomic knowledge or prior NGS knowledge, it has less restriction while more transferability (Poplin et al., 2017). In addition to Google, Deep Genomics is also dedicated to interrogate biological problems from the point of artificial intelligence, for instance, identifying disease-related mutations from large available data sets of genomics (Mesko, 2017).

### Neoantigen Discovery

The precise description of precision medicine is “an emerging approach for disease treatment and prevention that takes into account the individual variability in genes, environment and lifestyle for each person,” according to the Precision Medicine Initiative. One of such therapeutic methods is neoantigen-based immunotherapy. As the name suggests, neoantigens are practically antigens which are encoded specifically by the mutated genes in tumor cells (Lu and Robbins, 2016). Currently, three major classifications of tumor antigens have been recognized, they are separately tumor-specific antigens, tumor-associated antigens and cancer-germline/cancer testis antigens (Gubin et al., 2015). Among these different kinds of tumor antigens, neoantigens belong to the tumor-specific antigen class, which are only presented in tumor cells and thus would circumvent on-target but off-tumor reactivity (Bethune and Joglekar, 2017). What’s more, because neoantigens are tumor-specific, the central thymic tolerance would be bypassed (Ott et al., 2017). Back in 2003, Lawrence A. and co-workers first provided the evidence that single malignant cell within human tumors could harbor thousands of randomly generated mutations and these mutations formed a mutator phenotype, which finally resulted in the heterogeneity of human tumors (Loeb et al., 2003). Nevertheless, not all random mutations produced by tumor cells could finally become neoantigens. Among human cancer types, three tumors with the highest frequencies of somatic mutations are melanoma, lung squamous and lung adenocarcinoma (Alexandrov et al., 2013; Martincorena and Campbell, 2015). Strikingly, most melanomas have up to 10 somatic mutations for every megabase of coding DNA, which indicates approximately 150 non-synonymous mutations for expressed genes (Schumacher and Schreiber, 2015). From this point, it is sufficient to give rise to frequent formation of neoantigens. However, only part of the tumors could produce such high frequencies of somatic mutations. Some cancers like acute myeloid leukemia (AML) and acute lymphoblastic leukemia, have low mutational frequencies (Alexandrov et al., 2013; Martincorena and Campbell, 2015). Non-synonymous mutations within tumor genome are prerequisite for successful response. The corresponding mutated proteins translated by those mutations were first processed by proteasome into short peptides with 8–11 residues, then these peptides were transported into endoplasmic reticulum with the help of the transporter associated with antigen processing (Editorial, 2017). Once shuttled into the ER, these peptides would be possible to bind with the major histocompatibility complex class I (MHC-I), hence could be recognized by CD8+ T cells (Bethune and Joglekar, 2017). In brief, only those mutated peptides which could bind with MHC with high affinity and be recognized by T cells, and finally trigger immune response, can be termed as neoantigens.

In the past two decades, most of the discovered unique neoantigens were identified through cDNA library screening, such as the majority of neoantigens for melanoma, which were summarized by Lu and Robbins (2016). This classical labor-intensive screening method with low throughput was gradually giving way to the high-throughput sequencing methods, for example, the WES and RNA sequencing, with the development of computing power and sequencing technologies. A typical genomics-based identification framework for neoantigens comprises four steps. Firstly, tumor cell samples, as well as normal tissue, are subjected to the WES to identify those non-synonymous mutations and sometimes are also subjected to RNA sequencing to determine the antigen abundance. Then, the specific MHC subtypes of patient’s T cells are determined through PCR-based HLA sequencing (Hosomichi et al., 2015). Once completed, the responding mutant epitopes are analyzed and predicted for binding activity with specific MHC subtypes using *in silico* approaches to prioritize a list of candidate epitopes. Finally, the predicted peptides are synthesized and tested for relating phenotypes using experimental assays. More comprehensive descriptions could be found in many excellent reviews (Gubin et al., 2015; Schumacher and Schreiber, 2015; Hundal et al., 2016; Bethune and Joglekar, 2017). During the process of developing neoantigens, the accurate prediction of mutation associated neoantigens using computational methods is of great significance, which could accelerate the whole process and hence save much time and money. To embrace challenges of current computer-based neoantigen prediction, up to 30 universities, companies and those non-profit institutions got together, and co-founded an alliance called Tumor Neoantigen Selection Alliance (Editorial, 2017). Once a better prediction algorithm was established, neoantigen-based immunotherapy would galvanize a new wave of precision medicine.

The human MHC is synonymous with human leukocyte antigen complex (HLA), which can be divided into three classes. Amongst, MHC-I can be recognized by CD8 co-receptors of T cells, while MHC-II can be recognized by CD4 co-receptors (Janeway et al., 2001). Moreover, MHC-I, also known as HLA-I, is extremely polymorphic, with more than ten thousand alleles been discovered^2^. As far back as 2003, Morten and co-workers used ANN approach to quantitatively predict the binding affinity of peptides with HLA-A^∗^0204 (Nielsen et al., 2003). Then in 2007, with the accumulation of peptide-binding data and the prevalence of machine learning, they also developed the NetMHCpan, a prediction program for peptide binding to any HLA of known sequences (Nielsen et al., 2007; **Table 2**). They exploited both the information of peptide sequences and the residues of HLAs within 4.0 Å of the binding nanomer peptides, to construct an ANN with only one hidden layer. After training and optimizing the ANN model, NetMHCpan could achieve good prediction results. Of note, NetMHCpan was not intended to predict possible neoantigens at that time, though it is now used to perform peptide-binding prediction as part of the pipeline for neoantigen discovery and design. The latest version can be found here^3^. In 2017, a remarkable clinical research about personal neoantigen vaccine reported that four out of six vaccinated patients with melanoma had no recurrence within 25 months, and the other two patients who underwent disease recurrence then received anti-PD-1 therapy and finally achieved complete tumor regression (Ott et al., 2017). This work demonstrated the safety and feasibility of neoantigen-based vaccine, as well as the high efficiency of high-throughput sequencing combined with neoantigen prediction using *in silico* methods like NetMHCpan.

With the renaissance of deep learning in recent years, whether it could be used to predict peptide binding to MHCs has drawn wide attention, especially because of the potential application of tumor neoantigen. Researchers from Johns Hopkins University have developed five machine learning approaches, including MHCnuggets-GRU, MHCnuggets-LSTM and three CNN-based prediction methods, to predict peptide binding affinity to MHC-I (Bhattacharya et al., 2017). In the meantime, they conducted a benchmark study to compare these methods with existing peptide prediction approaches like NetMHC (Lundegaard et al., 2008) and NetMHCpan (Nielsen et al., 2007). It turned out that both GRU and LSTM-based models could achieve equivalent accuracy. MHCnuggets-GRU and MHCnuggets-LSTM are RNN-based deep learning algorithms. They both comprise a fully connected layer with 64 hidden units and are regularized with a recurrent dropout. Another research group from Stanford University School of Medicine has trained a prediction program named Maria for accurate prediction of peptide binding with MHC-II through RNN-based deep learning algorithm (Chen et al., 2017). During the training process, they incorporated several features including peptide sequences, specific context within each protein, gene expression data and patient MHC alleles to build the datasets for training Maria.

The advancement of computing power especially accelerated GPU as well as the great breakthrough of deep learning algorithms has made artificial intelligence become an active research area. Increasingly accumulated biological data like clinical samples and high-throughput sequencing data provides massive information while deep learning approaches could make full use of these data to find potential connections, which could be of great importance for precision medicine. In addition to the analysis of genetic variants and neoantigen prediction, deep learning could also be helpful in predicting possible metabolic sites of drugs and the polymorphism of metabolic enzymes to provide basis for clinical medication (Hughes et al., 2015; Lauschke and Ingelman-Sundberg, 2016). Nevertheless, some limitations still exist: current databases about systematic clinical information are still deficient and public accessible data is unable to completely satisfy the demand of artificial intelligence. In addition, the complexity of biological system makes prediction of potential function based on existing data more elusive. Whether it is a hit-or-miss affair for using artificial intelligence to solve biological problems remains to be seen.

## Antibody-Drug Conjugates (ADC)

**FIGURE 9:**
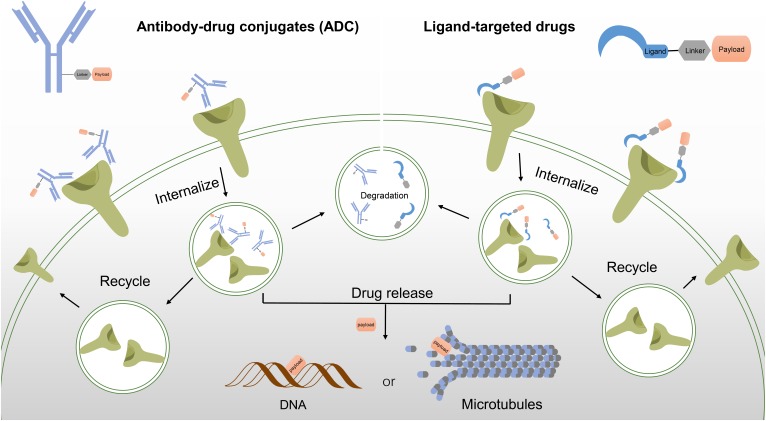
Antibody-drug conjugates (ADC) and ligand-targeted drugs share similar mechanism in disrupting cellular functions.

In the development of ADCs, a monoclonal antibody which targets on a specific antigen expresses in certain cancer cells is often conjugated by a highly potent cytotoxic payload via a suitable linker (Chari et al., 2014). The binding of ADCs to the antigen targets on the cell surface will initiate the internalization of the ADC-antigen complex. Upon entering the cell, the warheads bind to their target and disrupt the cellular function, which lead to irreversible cell death. General processes including antibody internalization, degradation or recycling and drug release are illustrated as **Figure 9**.

### First Generation ADCs

Gemtuzumab ozogamicin (Mylotarg, Wyeth/Pfizer) is a typical example of the first generation ADCs that suffer from the heterogeneity problem and toxicity issues (Parigger et al., 2016). Mylotarg consists of a monoclonal antibody targeted against the CD33 antigen present on myeloblastic leukemia cells and a cytotoxic calicheamicin derivative with a cleavable hydrazone linker. It had achieved market approval under an accelerated approval program by FDA in 2000 (Zuboy, 2000). However, it failed to demonstrate its clinical endpoint benefit and met with worrisome fatal toxicity rate in post marketing surveillance acquired by FDA. Therefore, Mylotarg was withdrawn from the market in 2010 (Lancet, 2013). Strikingly, after three open-label clinical studies which lowered the administration dose and frequency that confirmed its clinical benefit, Mylotarg was back to the market for treating patients with newly diagnosed CD33-positive AML or relapsed or refractory CD33-positive AML in September 1, 2017. The returning of Mylotarg brings hopes to patients suffered from these notorious diseases. However, the noticeable hepatotoxicity, especially venoocclusive disease should also be seriously considered as a result of that FDA has required a black box warning in the prescribing information of Mylotarg (Hedrich et al., 2017).

The toxic side effects in patients treated with Mylotarg may be partly owing to the fact that the CD33 antigen target shares expression on myeloid progenitor cells, thus affecting the normal function of these cells and causing myelosuppression, such as neutropenia and thrombocytopenia (Lancet, 2013). Moreover, patients with CD33-negative leukaemias also showed response to Mylotarg. One reason may ascribe the CD33-independent mechanism that internalizing drugs without antigen and antibody reaction, and another may attribute to the unstable hydrazone linker which is liable to hydrolysis and likely to release of its warhead in blood circulations (Singh and Erickson, 2009). In addition, since the drug-linker moiety is attached to the random lysines on the mono antibody, Mylotarg comprises 1–8 cytotoxic payloads per IgG molecule as well as ∼50% naked antibody which fails to conjugate any warheads (Jawad et al., 2010). Moreover, Mylotarg selects a humanized antibody of IgG4 isotype which is likely to form half antibodies (one heavy and one light chain being linked) *in vivo* and has limited ability to exhibiting secondary immune functions, such as antibody-dependent cell-mediated cytotoxicity (ADCC) and complement-dependent cytotoxicity (Villamor et al., 2003). These poor characteristics in heterogeneity and quality control have made Mylotarg a formidable and risky drug in the long-term clinical development. However, the second approval of Mylotarg by FDA has confirmed the clinical benefit for treatment of CD33-positive AML, thus giving confidence to the whole field of ADCs (Daver et al., 2016).

### Second Generation ADCs

Trastuzumab emtansine (also known as T-DM1 and Kadcyla, developed by Genentech/Roche) compromises a microtubule polymerization inhibitor DM1 that is conjugated via a stable thioether linker to a humanized IgG1 anti-HER2 mAbs (Doroshow and LoRusso, 2017). In 2013, T-DM1 was granted specifically by FDA for treatment of HER2-positive metastatic breast cancer (Amiri-Kordestani et al., 2014). HER2 is a member of the human epidermal growth factor receptor family, and its overexpression has been reported in various cancer types, especially human breast cancers (∼15–30%) and gastric cancers (7–34%) (Iqbal and Iqbal, 2014). In recent years, HER2 has become an important biomarker and target for diagnosing and treating HER2-positive breast cancer patients. The early approved drug Trastuzumab (Herceptin, Roche) has shown adequate activity against HER2 receptors and was then used to develop Trastuzumab emtansine that linked to a derivative of maytansine which was too toxic when used as a free cytotoxic agent (Gemmete and Mukherji, 2011). As shown from a Phase III clinical trial (EMILIA) that compared the safety and efficacy of T-DM1 with the combination therapy of Capecitabine and Lapatinib in participants whose conditions could not be controlled by Trastuzumab alone, T-DM1 exhibited promising clinical activity and could prolong the survival by at least 5 months of patients with advanced breast cancer (Welslau et al., 2014). However, in another 3-arm, phase III study (MARIANNE) that compared T-DM1 with or without Pertuzumab versus Trastuzumab and Taxane therapy showed unsatisfied outcomes in consideration of taking T-DM1 as a first-line treatment solution of HER2-positive, advanced breast cancer [overall survival (OS): 50.86 months with trastuzumab plus taxane, 53.68 months with T-DM1, and 51.78 months with T-DM1 plus pertuzumab] (Perez et al., 2017). So far, T-DM1 is still active in several clinical trials, and most of them are combination therapies including T-DM1 with Pembrolizumab, Neratinib, or Tucatinib.

The main improvements of second generation ADCs are preferable drug quality controls and minimal toxicity effects. Though FDA has added a warning label including hepatotoxicity, cardiac toxicity and embryo-fetal toxicity to T-DM1 packaging, less patients suffered from severe toxic effects than who received standard therapy (lapatinib + capecitabine) in clinical trials (Sendur et al., 2013). The reason why T-DM1 shows less systemic toxicity may be partly due to its rather stable thiosuccinimide linker which is less prone to being cleaved under physiological conditions (Mohamed et al., 2018). However, it should be noted that the thiosuccinimide moiety could slowly undergo reversible transformation to thiols and alkyl maleimides through retro-Michael elimination reaction. This reaction may lead to cytotoxic drug loss and eventually compromise the efficacy of T-DM1 as well as cause off-target toxicity. In terms of the drug-to-antibody ratio (DAR), T-DM1 has an average DAR of 3.5 (from 0 to 8 linker-drug molecules per antibody) in aqueous formulation, while the unconjugated antibodies remain only 5% which is much better than the first generation ADCs which contains 50% naked antibodies (Kim et al., 2014).

Besides, most second generation ADCs select IgG1 isotype as their mAbs owing to its cost-effective manufacture and the ability to support ADCC (Marcoux et al., 2015). The targeted drug Trastuzumab alone could bind to HER2 receptors and recruit immune effector cells that actively lyse the target cells, and T-DM1 shares the same mechanisms of action. Notably, companies still argue about the ADCC effect, caused either by the naked antibodies or the IgG1 isotype, on whether ADCC enhances the efficacy or lowers the tolerance when in use (Chen et al., 2016). Nevertheless, the approved drug T-DM1 has provided a good example of balancing both the efficacy and safety for HER2-positive breast cancer patients.

Another second generation ADC that has been approved is Brentuximab vedotin (Adcetris, Seattle Genetics). Brentuximab vedotin has the same antibody isotype (IgG1) and shares the properties of heterogeneity as T-DM1 but differs in its protease-cleavable linker and the cytotoxic warheads which contains monomethyl auristatin E (MMAE) moieties (Gualberto, 2012). Brentuximab vedotin was designed to target the cell-membrane protein CD30 which often occurs on the surface of malignant lymphoma cells but rarely on that of normal cells. In November 2017, Brentuximab vedotin was granted approval for the treatment of adult patients with CD30-expressing mycosis fungoides (MF) or primary cutaneous anaplastic large cell lymphoma (pcALCL) who have received prior systemic therapy (Duvic et al., 2015). One of the main characteristics of Brentuximab vedotin is the peptide-based linker that attached to the hinge cysteines of the antibody. Once Brentuximab vedotin is internalized by the targeted cells, the vesicle fuses with lysosomes and the valine-citrulline linker is gradually cleaved by lysosomal cysteine proteases, especially cathepsin B (van de Donk and Dhimolea, 2012). As a result, the released drugs (MMAE) bind to the tubulin protein and disrupt the normal microtubule function, which leads to the final cancer cell death.

In summary, the second generation ADCs have improved stability and showed better CMC (chemistry, manufacturing and controls) characteristics owing to the improved linkers and the change of IgG1 isotype, but are still in a dilemma about the heterogeneity and systemic toxicity.

### Third Generation ADCS

Vadastuximab talirine (Seattle Genetics) delivers the cytotoxic payload specifically to malignant cells bearing CD33 antigens (Stein et al., 2017). Just like the first generation ADC gemtuzumab ozogamicin, it is designed for the treatment of AML or other hematological cancer including myelodysplastic syndrome. So far, there are five studies concerning the safety and efficacy of vadastuximab talirine with three studies terminated and no results posted. Compared with the second generation ADC Brentuximab vedotin, Vadastuximab talirine also contains a humanized IgG1 mAb and a protease-cleavable linker. However, it differs in the warhead structure which contains a pyrrolobenzodiazepine dimer that is optimized for overcoming multidrug resistance via the efflux pumps (Kung Sutherland et al., 2013). Notably, a breakthrough of Vadastuximab talirine is making use of site-specific technologies to engineer residue serine to residue cysteine (S239C) on both heavy chains. This site-specific conjugation significantly increases the homogeneity of Vadastuximab talirine, making it a uniform product with average DAR of 2 in aqueous solution. Vadastuximab talirine has demonstrated the efficacy and tolerability in AML animal models, while the clinical benefit is yet to be seen (Borthakur, 2013).

In addition to the site-specific technologies, other state-of-the-art technologies have also been used in the development of third generation ADCs (Beck et al., 2017). Technologies including unnatural amino acid engineering, native cysteine rebridging, glycoconjugation, enzyme-assisted ligation have been well discussed in several recent reviews. A summary of the first, second and third ADCs characteristics are illustrated in **Table 3**.

**Table 3:** Detailed information on antibody-drug conjugates (ADCs) and ligand-targeted drugs.

Name	Target	Linker	Payload	Indication	Current stage
**Antibody–drug conjugates (ADCs)**					
Gemtuzumab ozogamicin	CD33	Cleavable hydrazone linker attached to random lysines	Calicheamicin	Acute myeloid leukemia	Approved by FDA for the treatment of newly diagnosed CD33-positive acute myeloid leukemia in 2017
Trastuzumab emtansine	HER2	Non-cleavable thioether linker attached to random lysines	DM1	HER2^+^ metastatic breast cancer	Entered market in 2013
Brentuximab vedotin	CD30	Protease-cleavable linker attached to hinge cysteines	MMAE	Anaplastic large-cell lymphoma and Hodgkin lymphoma	Entered market in 2013
Vadastuximab talirine	CD33	Protease-cleavable linker attached to engineered heavy-chain cysteine (S239C)	SGD-1882	Acute myeloid leukemia	PhaseIII for acute myeloid leukemia
**Ligand-targeted therapeutic agents**					
177Lu-PSMA617	PSMA	By labeling	^177^Lu	Prostate cancer	PhaseII in progressive metastatic castration resistant prostate cancer
Vintafolide	FR	Disulphide bond linker	Vinblastine	Ovarian cancer; endometrial cancer; adenocarcinoma of the lung; solid tumor; non-small cell lung cancer	PhaseI for ovarian cancer, endometrial cancer has been completed; Phases I and II for non-small cell lung cancer, solid tumor have been completed
**Ligand-targeted imaging agents**					
Etarfolatide	FR	Peptide linker	Technetium-99m	FR-positive malignant diseases (e.g., lung, kidney, brain or ovarian cancer); autoimmune diseases	Phase III in FR-positive cancer
OTL38	FR	peptide linker	NIR dye	Fluorescence-guided surgery	PhaseIII for fluorescence-guided surgery of ovarian, breast, lung and kidney cancers

However, whether the aforementioned novel technologies could assist in the development of more potent, stable, homogenous and tolerable ADCs is eagerly expected by both academia and industry as well as doctors and patients who count on novel treatment methods and technologies to extend their lives.

## Ligand-Targeted Drugs

Unlike antibody drug conjugates, ligand-targeted drugs employ a target ligand with an appropriate linker to deliver the attached payloads to the desired pathologic cell (**Figure 9**). Many aspects have to be considered in design of ligand-targeted drugs including the targeting ligand (concerning an appropriate cell target), the proper linker and the toxic payloads (Srinivasarao and Low, 2017).

Firstly, the target should be specific to the chosen conditions relative to normal cells so that it can guarantee the safety of the conjugates and control the toxicity from the unspecific binding (Muro, 2012). Besides, the absolute number of targets (usually receptors) should be adequate for the conjugates to bind so that the potency can be assured. In addition, in terms of ligand selection, the most important factor is the binding affinity. The dissociation constant (*K*_d_) of a ligand for its receptor should be approximately 10 nM or lower so that a desired therapeutic effect may be achieved. Also, the coupling between the ligand and the linker should have little influence on the ability of ligands to bind to their receptors (Leamon et al., 2014).

Secondly, an optimal linker should attach both the ligand and the payload in a steric proper manner. In addition, the linker should have good properties of both pharmacokinetics and pharmacodynamics and it is even better to be efficiently cleavable within cells (Kurzrock et al., 2012). Especially for the therapeutic agents, linkers that contain disulphide bond (reduced by intracellular glutathione (GSH)) or peptide-based linker (cleaved by cathepsin B in lysosomes) or even self-cleaving linkers, including hydrazones and ester linkers (hydrolysed at low pH in endosomes) are very popular in designing intracellular release drugs (Vlahov and Leamon, 2012).

Thirdly, the cytotoxic payloads should have a high potency with an IC_50_ of at least 10 nM, and preferably pM. In addition to the high potency, the drugs should have a low molecular weight, and easy derivatizability as well as good membrane permeability (Erez et al., 2009). Although the ligand-drug conjugates enter the cell via receptor-mediated endocytosis, the drugs upon being cleaved within endosomes still have to pass across lipid bilayers.

All in all, the requirements for each part of ligand-targeted drugs are very strict. Several key factors related to potency, selectivity and physicochemical properties should be carefully optimized in order to achieve a successful conjugate (**Table 3**). Following are several typical examples of ligand-targeted drugs both for treatment and diagnosis application.

## Ligand-Targeted Therapeutic Agents

### 177Lu-PSMA617

177Lu-PSMA617 represents as a novel radioligand therapy for treatment of metastatic castration resistant prostate cancer (Afshar-Oromieh et al., 2015). Traditional beam radiation therapy often prevents tumor growth or its recurrence after surgery by using ionizing radiation through a linear accelerator. The ionizing radiation can damage the DNA of cancer cells which finally results in cellular death, however, it hard to bypass the surrounding healthy tissues. 177Lu-PSMA617 provides an approach via linkage of a radioisotope to a targeting ligand that can deliver the radioactive element specifically to the cancerous cells. The ligand of 177Lu-PSMA617 anchors on prostate specific membrane antigen (PSMA), which is a type II transmembrane protein that expressed highly on most prostate cancer cells but absent on most normal tissues (Afshar-Oromieh et al., 2016). It serves as an important biomarker in clinic and versatile diagnostic tools that are commercially available. 177Lu-PSMA617 utilizes a highly affinity ligand targeting on PSMA and directing the radioactive atom Lutetium 177 to kill prostate cancer cells (Kratochwil et al., 2016). Lutetium-177 (177Lu) delivers energetic beta particles to the targeting cells that express PSMA while sparing the healthy ones (Chakraborty et al., 2016). It should be noted that once 177Lu-PSMA617 is injected, it travels through the blood circulation and could target multiple sites of prostate cancer cells, even including the metastatic cells. So far, 177Lu-PSMA617 has been allowed to an open-label, phase 2 trial which aims to determine the efficacy and safety of 177Lu-PSMA617 compared to cabazitaxel in patients with progressive metastatic castration resistant prostate cancer (Das et al., 2016). Prostate-specific antigen response rate, progression free survival, pain response and adverse events will be recorded and the correlation between positron-emission tomography (PET) imaging and clinical benefit will be investigated.

### Vintafolide

177Lu-PSMA617 is developed by a biopharmaceutical company, Endocyte, which makes great efforts in building platform for small molecule drug conjugates. Several ligand-targeted drugs developed by Endocyte have entered clinical trials. Most of the drugs aim at treating solid tumors and are constructed of a small molecule targeting folate receptor. Vintafolide is a typical example and it consists of a ligand targeting the folate receptor and a cytotoxic chemotherapy payload, vinblastine. Folate receptor is overexpressed on certain aggressively growing cancers including ovarian cancers, non-small lung cancers, and renal cell carcinoma (Graybill and Coleman, 2014). Folate, also known as vitamin B9, has already been approved by FDA to treat anemia and to prevent NTD during pregnancy. Once folate-vinblastine binds to the folate receptor, it is subsequently internalized through endocytosis process and releases the drug to kill cancer cells (Vergote and Leamon, 2015). Vintafolide has been tried in several aggressive cancers but failed to meet with primary endpoint of progression-free survival (Luyckx et al., 2014). The poor metabolic stability may contribute to the low efficacy in clinical trials, and since there is no disclosed data concerning safety problems, cautions should be held for such types of conjugated drugs. In fact, most of the ligand-targeted therapeutic agents developed have been questioned by clinical trials. Agents include EC0225 (folate-mitomycin C), EC0489 (folate-vinblastine), EC17 (folate-FITC-hapten) have been halted owing to the unsatisfied clinical outcomes (Amato et al., 2013). However, an exploratory study of the folate-tubulysin drug conjugates EC1456 in ovarian cancer and a phase IA/B study in patients with advanced solid tumors are still ongoing (Vergote et al., 2015). Taken together, whether the ligand-targeted therapeutic agents could play a role in future precision medicine remains to be seen.

## Ligand-Targeted Imaging Agents

In the era of individualized medicine, ligand-targeted imaging agents have been appropriately matched with various kinds of therapeutic targeting drugs. Several agents have shown promising clinical outcomes in diagnosis thus benefiting both doctors and patients for choosing the appropriate targeting drugs. Following is two typical ligand-targeted agents that developed to diagnose cancers or arthritis diseases.

### Technetium (99mTc) Etarfolatide

Unlike ligand-targeted therapeutic agents, the imaging agents from Endocyte company consist of a small molecule targeting the folate receptor and an imaging item named technetium-99m (Maurer et al., 2014). Technetium-99m (99mTc) is a commonly used radioactive tracer which can emit detectable gamma rays acquired by the medical equipment. Of note, the physical half-life of 99mTc is relative short, which restricts its role in therapeutic use but highlights its application in diagnosis.

Strikingly, in addition to the cancers that have high levels of folate expression, autoimmune diseases such as arthritis, multiple sclerosis and psoriasis which harbor activated macrophages can also be detected by 99mTc etarfolatide. This utilization is based on the principle that the expression of folate receptor is selectively elevated on activated macrophages but not dormant ones (Xia et al., 2009). And macrophages are only activated at the site of inflammation owing to autoimmune disease or injury. Technetium (99mTc) etarfolatide was originally designed to be a companion of ligand-targeted therapeutic agents, and has been investigated in several clinical trials for identification of its biodistribution as well as its safety and efficacy (Morris et al., 2014). In a clinical trial that aimed to determine whether 99mTc etarfolatide could target sites of inflammation in the joints or organs in patients with autoimmune diseases, several patients showed obvious 99mTc etarfolatide uptake in their multiple joints of hands and feet (Xia et al., 2009). This clinical trial has provided the evidence that folate-conjugated imaging agents could detect the inflammation area where activated macrophages are enriched in patients with rheumatoid., since the ligand-targeted therapeutic agents have failed to confirm its efficacy in clinic, the further development of 99mTc etarfolatide has also been halted.

### OTL38

OTL38, like Technetium (99mTc) etarfolatide, contains a folate receptor-targeting ligand which is specially used for imaging of folate receptor overexpressing tumors (Kelderhouse et al., 2013). OTL38 consists of a fluorescent near infrared (NIR) dye which allows for the easy visualization of fluorescent tumor cells (Lee et al., 2017). This folate receptor-targeted NIR probe is aimed at assisting surgeons in identification of the malignant lesions and increasing the successful rate when performing complete surgical resection (Predina et al., 2017b). OTL38 is currently being developed in several clinical trials, including a phase 3 ovarian cancer study (with safety already be tested in a phase 2 study), and a phase 2 exploratory study of identifying pulmonary nodules (van Dam et al., 2011; Hoogstins et al., 2016). Moreover, treatments of additional cancer types including pituitary neoplasms, renal cell carcinoma, bladder cancer and gastric cancer are also undergoing clinical trial for testing OLT38’s safety and efficacy (Predina et al., 2017a, 2018).

In summary, although currently no product of ligand-targeted drugs has entered into the market, it still holds great hope for the development of these conjugates for treatment and especially diagnosis of patients with specific biomarker (Assaraf et al., 2014). Together with ADCs, they will serve as precise powerful weapons for certain diseases, especially cancer.

## Further Perspective

The discovery of potent and selective modulators will continue to be central to revealing the physiological functions of isozymes and to the development of new therapeutic or diagnostic agents for many diseases. In this review, we focused on strategies and applications for precision drug design. SBDD, computational modeling and simulation approaches, deep learning based genetic variant discovery and neoantigen prediction, ADCs, and ligand-targeted conjugate drugs, as well as ligand-targeted imaging agents are discussed in detail. Through combination of various kinds of strategies, novel therapeutic strategies based on precision drug design are highly expected. Recently, screening for inhibitor specificity using multiprotein complexes rather than purified proteins or the addition of requisite protein-protein interaction assay is advocated. Strikingly, a comprehensive understanding of the complex physiological function of isoforms and cell-type specific protein complexes with their substrates and modulators becomes essentially important in the design of highly potent and selective inhibitors.

With the increasingly fundamental role of precision medicine in drug discovery and development, it could benefit for both targeted drug design and clinical trial design. In the meantime, personalized approach provides new challenges for better consideration of biomarker related clinical indication. In addition to the various targeted drug design strategies, precision immunotherapy approaches especially monoclonal antibodies targeted tumor-specific antigens, cell-based therapy like chimeric antigen receptor (CAR) T cell therapy, and vaccines are also important for precision therapies. In addition, various drug combinations could make great contributes to overcoming drug resistance and providing better efficacy. We believe that by integrating all kinds of strategies, it is promising to design highly selective bioactive molecules for specific molecular recognition in complex biological systems and will ultimately provide precision medicine beneficial for human.

## Author Contributions

All authors made significant contributions to the preparation of the manuscript and approved it before submission. CL and KZ developed the paper design, and finalized the manuscript. CW and PX wrote the manuscript, edited the references, and revised the style of the manuscript. JH and LZ helped to collect the data, complete the table, and revise the manuscript.

## Conflict of Interest Statement

The authors declare that the research was conducted in the absence of any commercial or financial relationships that could be construed as a potential conflict of interest.

## Footnotes

1 https://obamawhitehouse.archives.gov/node/333101

2 https://www.ebi.ac.uk/ipd/imgt/hla

3 http://www.cbs.dtu.dk/services/NetMHCpan/

## Abbreviations

ADCs: antibody-drug conjugates
ANN: artificial neural network
CA-4: combretastatin A-4
CNN: convolutional neural network
CTD: C-terminal kinase domain
DFSA: 2,3-difluorosialic acid
DNN: deep neural network
GPU: graphic processing units
HTS: high-throughput screening
NGS: next-generation sequencing
NTD: neural tube defects
PCPA: *trans*-2-phenyl-cyclopropylamine
RNN: recurrent neural network
SBDD: structure-based drug design
SNP: single nucleotide variants
WES: whole-exome sequencing
WGS: whole-genome sequencing.

## References

[B1] Afshar-Oromieh A. Babich J. W. Kratochwil C. Giesel F. L. Eisenhut M. Kopka K. (2016). The rise of PSMA ligands for diagnosis and therapy of prostate cancer. *J. Nucl. Med.* 57 79s–89s. 10.2967/jnumed.115.170720 27694178

[B2] Afshar-Oromieh A. Hetzheim H. Kratochwil C. Benesova M. Eder M. Neels O. C. (2015). The theranostic PSMA ligand PSMA-617 in the diagnosis of prostate cancer by PET/CT: biodistribution in humans, radiation dosimetry, and first evaluation of tumor lesions. *J. Nucl. Med.* 56 1697–1705. 10.2967/jnumed.115.161299 26294298

[B3] Alexandrov L. B. Nik-Zainal S. Wedge D. C. Aparicio S. A. Behjati S. Biankin A. V. (2013). Signatures of mutational processes in human cancer. *Nature* 500 415–421. 10.1038/nature12477 23945592 PMC3776390

[B4] Amato R. J. Shetty A. Lu Y. Ellis R. Low P. S. (2013). A phase I study of folate immune therapy (EC90 vaccine administered with GPI-0100 adjuvant followed by EC17) in patients with renal cell carcinoma. *J. Immunother.* 36 268–275. 10.1097/CJI.0b013e3182917f59 23603861

[B5] Amiri-Kordestani L. Blumenthal G. M. Xu Q. C. Zhang L. Tang S. W. Ha L. (2014). FDA approval: ado-trastuzumab emtansine for the treatment of patients with HER2-positive metastatic breast cancer. *Clin. Cancer Res.* 20 4436–4441. 10.1158/1078-0432.ccr-14-0012 24879797

[B6] Asaba T. Suzuki T. Ueda R. Tsumoto H. Nakagawa H. Miyata N. (2009). Inhibition of human sirtuins by in situ generation of an acetylated lysine-ADP-ribose conjugate. *J. Am. Chem. Soc.* 131 6989–6996. 10.1021/ja807083y 19413317

[B7] Assaraf Y. G. Leamon C. P. Reddy J. A. (2014). The folate receptor as a rational therapeutic target for personalized cancer treatment. *Drug Resist. Updat.* 17 89–95. 10.1016/j.drup.2014.10.002 25457975

[B8] Bamborough P. Brown M. J. Christopher J. A. Chung C. W. Mellor G. W. (2011). Selectivity of kinase inhibitor fragments. *J. Med. Chem.* 54 5131–5143. 10.1021/jm200349b 21699136

[B9] Baskin I. I. Winkler D. Tetko I. V. (2016). A renaissance of neural networks in drug discovery. *Expert Opin. Drug Discov.* 11 785–795. 10.1080/17460441.2016.1201262 27295548

[B10] Beck A. Goetsch L. Dumontet C. Corvaia N. (2017). Strategies and challenges for the next generation of antibody-drug conjugates. *Nat. Rev. Drug Discov.* 16 315–337. 10.1038/nrd.2016.268 28303026

[B11] Bethune M. T. Joglekar A. V. (2017). Personalized T cell-mediated cancer immunotherapy: progress and challenges. *Curr. Opin. Biotechnol.* 48 142–152. 10.1016/j.copbio.2017.03.024 28494274

[B12] Bhattacharya R. Sivakumar A. Tokheim C. Guthrie V. B. Anagnostou V. Velculescu V. E. (2017). Prediction of peptide binding to MHC Class I proteins in the age of deep learning. *bioRxiv* [Preprint]. 10.1101/154757

[B13] Biesecker L. G. Spinner N. B. (2013). A genomic view of mosaicism and human disease. *Nat. Rev. Genet.* 14 307–320. 10.1038/nrg3424 23594909

[B14] Boehm J. S. Hahn W. C. (2011). Towards systematic functional characterization of cancer genomes. *Nat. Rev. Genet.* 12 487–498. 10.1038/nrg3013 21681210

[B15] Borthakur G. (2013). Precision ‘re’arming of CD33 antibodies. *Blood* 122:1334. 10.1182/blood-2013-06-509638 23970353

[B16] Carter T. C. He M. M. (2016). Challenges of identifying clinically actionable genetic variants for precision medicine. *J. Healthc. Eng.* 2016:3617572. 10.1155/2016/3617572 27195526 PMC4955563

[B17] Celada F. Seiden P. E. (1992). A computer model of cellular interactions in the immune system. *Immunol. Today* 13 56–62. 10.1016/0167-5699(92)90135-T 1575893

[B18] Celada F. Seiden Philip E. (1996). Affinity maturation and hypermutation in a simulation of the humoral immune response. *Eur. J. Immunol.* 26 1350–1358. 10.1002/eji.1830260626 8647216

[B19] Chakraborty S. Chakravarty R. Shetty P. Vimalnath K. V. Sen I. B. Dash A. (2016). Prospects of medium specific activity (177) Lu in targeted therapy of prostate cancer using (177) Lu-labeled PSMA inhibitor. *J. Labelled Comp. Radiopharm.* 59 364–371. 10.1002/jlcr.3414 27264278

[B20] Chari R. V. Miller M. L. Widdison W. C. (2014). Antibody-drug conjugates: an emerging concept in cancer therapy. *Angew. Chem. Int. Ed. Engl.* 53 3796–3827. 10.1002/anie.201307628 24677743

[B21] Chen B. Michael K. Niclas O. Ethan F. Lisa E. W. Liu C. L. (2017). Maria: accurate prediction of MHC-II peptide presentation with deep-learning and lymphoma patient MHC-II ligandome. *Blood* 130:1486.

[B22] Chen L. Wang L. Shion H. Yu C. Yu Y. Q. Zhu L. (2016). In-depth structural characterization of Kadcyla(R) (ado-trastuzumab emtansine) and its biosimilar candidate. *MAbs* 8 1210–1223. 10.1080/19420862.2016.1204502 27380163 PMC5058630

[B23] Choi M. Scholl U. I. Ji W. Liu T. Tikhonova I. R. Zumbo P. (2009). Genetic diagnosis by whole exome capture and massively parallel DNA sequencing. *Proc. Natl. Acad. Sci. U.S.A.* 106 19096–19101. 10.1073/pnas.0910672106 19861545 PMC2768590

[B24] Cohen M. S. Zhang C. Shokat K. M. Taunton J. (2005). Structural bioinformatics-based design of selective, irreversible kinase inhibitors. *Science* 308 1318–1321. 10.1126/science1108367 15919995 PMC3641834

[B25] Congreve M. Langmead C. J. Mason J. S. Marshall F. H. (2011). Progress in structure based drug design for G protein-coupled receptors. *J. Med. Chem.* 54 4283–4311. 10.1021/jm200371q 21615150 PMC3308205

[B26] Copeland R. A. Pompliano D. L. Meek T. D. (2006). Drug-target residence time and its implications for lead optimization. *Nat. Rev. Drug Discov.* 5 730–739. 10.1038/nrd2082 16888652

[B27] Das T. Guleria M. Parab A. Kale C. Shah H. Sarma H. D. (2016). Clinical translation of (177)Lu-labeled PSMA-617: Initial experience in prostate cancer patients. *Nucl. Med. Biol.* 43 296–302. 10.1016/j.nucmedbio.2016.02.002 27150032

[B28] Daver N. Kantarjian H. Ravandi F. Estey E. Wang X. Garcia-Manero G. (2016). A phase II study of decitabine and gemtuzumab ozogamicin in newly diagnosed and relapsed acute myeloid leukemia and high-risk myelodysplastic syndrome. *Leukemia* 30 268–273. 10.1038/leu.2015.244 26365212 PMC4790089

[B29] Doroshow D. B. LoRusso P. M. (2017). Trastuzumab emtansine: determining its role in management of HER2 + breast cancer. *Future Oncol.* 14 589–602. 10.2217/fon-2017-0477 29214842

[B30] Drachman J. G. Senter P. D. (2013). Antibody-drug conjugates: the chemistry behind empowering antibodies to fight cancer. *Hematology Am. Soc. Hematol. Educ. Program* 2013 306–310. 10.1182/asheducation-2013.1.306 24319196

[B31] Dror R. O. Green H. F. Valant C. Borhani D. W. Valcourt J. R. Pan A. C. (2013). Structural basis for modulation of a G-protein-coupled receptor by allosteric drugs. *Nature* 503 295–299. 10.1038/nature12595 24121438

[B32] Duvic M. Tetzlaff M. T. Gangar P. Clos A. L. Sui D. Talpur R. (2015). Results of a phase II trial of brentuximab vedotin for CD30 + cutaneous T-cell lymphoma and lymphomatoid papulosis. *J. Clin. Oncol.* 33 3759–3765. 10.1200/jco.2014.60.3787 26261247 PMC4737859

[B33] Editorial (2017). The problem with neoantigen prediction. *Nat. Biotechnol.* 35:97. 10.1038/nbt.3800 28178261

[B34] Ekambaram R. Enkvist E. Vaasa A. Kasari M. Raidaru G. Knapp S. (2013). Selective bisubstrate inhibitors with sub-nanomolar affinity for protein kinase Pim-1. *ChemMedChem.* 8 909–913. 10.1002/cmdc.201300042 23616352

[B35] Erez R. Segal E. Miller K. Satchi-Fainaro R. Shabat D. (2009). Enhanced cytotoxicity of a polymer-drug conjugate with triple payload of paclitaxel. *Bioorg. Med. Chem.* 17 4327–4335. 10.1016/j.bmc.2009.05.028 19482477

[B36] Erlanson D. A. McDowell R. S. O’Brien T. (2004). Fragment-based drug discovery. *J. Med. Chem.* 47 3463–3482. 10.1021/jm040031v 15214773

[B37] Euan A. A. Atul J. B. Matthew T. W. Chen R. Teri E. K. Frederick E. D. (2010). Clinical assessment incorporating a personal genome. *Lancet* 375 1525–1535. 10.1016/s01406736(10)60599-5 20435227 PMC2937184

[B38] Fabre B. Ramos A. de Pascual-Teresa B. (2014). Targeting matrix metalloproteinases: exploring the dynamics of the s1’ pocket in the design of selective, small molecule inhibitors. *J. Med. Chem.* 57 10205–10219. 10.1021/jm500505f 25265401

[B39] Figge M. T. Garin A. Gunzer M. Kosco-Vilbois M. Toellner K. M. Meyer-Hermann M. (2008). Deriving a germinal center lymphocyte migration model from two-photon data. *J. Exp. Med.* 205 3019–3029. 10.1084/jem.20081160 19047437 PMC2605235

[B40] Fry D. W. Bridges A. J. Denny W. A. Doherty A. Greis K. D. Hicks J. L. (1998). Specific, irreversible inactivation of the epidermal growth factor receptor and erbB2, by a new class of tyrosine kinase inhibitor. *Proc. Natl. Acad. Sci. U.S.A.* 95 12022–12027. 10.1073/pnas.95.20.12022 9751783 PMC21758

[B41] Gawehn E. Hiss J. A. Schneider G. (2016). Deep learning in drug discovery. *Mol. Inform.* 35 3–14. 10.1002/minf.201501008 27491648

[B42] Gemmete J. J. Mukherji S. K. (2011). Trastuzumab (herceptin). *AJNR Am. J. Neuroradiol.* 32 1373–1374. 10.3174/ajnr.A2619 21816914 PMC7964332

[B43] Goodwin S. McPherson J. D. McCombie W. R. (2016). Coming of age: ten years of next-generation sequencing technologies. *Nat. Rev. Genet.* 17 333–351. 10.1038/nrg.2016.49 27184599 PMC10373632

[B44] Graybill W. S. Coleman R. L. (2014). Vintafolide: a novel targeted agent for epithelial ovarian cancer. *Future Oncol.* 10 541–548. 10.2217/fon.14.8 24754586

[B45] Gualberto A. (2012). Brentuximab Vedotin (SGN-35), an antibody-drug conjugate for the treatment of CD30-positive malignancies. *Expert Opin. Investig. Drugs* 21 205–216. 10.1517/13543784.2011.641532 22127011

[B46] Gubin M. M. Artyomov M. N. Mardis E. R. Schreiber R. D. (2015). Tumor neoantigens: building a framework for personalized cancer immunotherapy. *J. Clin. Invest.* 125 3413–3421. 10.1172/JCI80008 26258412 PMC4588307

[B47] Gupta A. Muller A. T. Huisman B. J. H. Fuchs J. A. Schneider P. (2017). Generative recurrent networks for *de novo* drug design. *Mol. Inform.* 37:1700111. 10.1002/minf.201700111 29095571 PMC5836943

[B48] Hagel M. Niu D. St Martin T. Sheets M. P. Qiao L. (2011). Selective irreversible inhibition of a protease by targeting a noncatalytic cysteine. *Nat. Chem. Biol.* 7 22–24. 10.1038/nchembio.492 21113170

[B49] Han Y. Gao S. Muegge K. Zhang W. Zhou B. (2015). Advanced applications of RNA sequencing and challenges. *Bioinform. Biol. Insights* 9 29–46. 10.4137/BBI.S28991 26609224 PMC4648566

[B50] Hedrich W. D. Fandy T. E. Ashour H. M. Wang H. Hassan H. E. (2017). Antibody-drug conjugates: pharmacokinetic/pharmacodynamic modeling, preclinical characterization, clinical studies, and lessons learned. *Clin. Pharmacokinet.* 57 687–703. 10.1007/s40262-017-0619-0 29188435 PMC6252075

[B51] Honigberg L. A. Smith A. M. Sirisawad M. Verner E. Loury D. Chang B. (2010). The Bruton tyrosine kinase inhibitor PCI-32765 blocks B-cell activation and is efficacious in models of autoimmune disease and B-cell malignancy. *Proc. Natl. Acad. Sci. U.S.A.* 107 13075–13080. 10.1073/pnas.1004594107 20615965 PMC2919935

[B52] Hoogstins C. E. Tummers Q. R. Gaarenstroom K. N. de Kroon C. D. Trimbos J. B. Bosse T. (2016). A novel tumor-specific agent for intraoperative near-infrared fluorescence imaging: a translational study in healthy volunteers and patients with ovarian cancer. *Clin. Cancer Res.* 22 2929–2938. 10.1158/1078-0432.ccr-15-2640 27306792

[B53] Hosomichi K. Shiina T. Tajima A. Inoue I. (2015). The impact of next-generation sequencing technologies on HLA research. *J. Hum. Genet.* 60 665–673. 10.1038/jhg.2015.102 26311539 PMC4660052

[B54] Howard S. Berdini V. Boulstridge J. A. Carr M. G. Cross D. M. Curry J. (2009). Fragment-based discovery of the pyrazol-4-yl urea (AT9283), a multitargeted kinase inhibitor with potent aurora kinase activity. *J. Med. Chem.* 52 379–388. 10.1021/jm800984v 19143567

[B55] Huang D. Caflisch A. (2011). Small molecule binding to proteins: affinity and binding/unbinding dynamics from atomistic simulations. *ChemMedChem* 6 1578–1580. 10.1002/cmdc.201100237 21674810

[B56] Hughes T. B. Miller G. P. Swamidass S. J. (2015). Modeling epoxidation of drug-like molecules with a deep machine learning network. *ACS. Cent. Sci.* 1 168–180. 10.1021/acscentsci.5b00131 27162970 PMC4827534

[B57] Hundal J. Miller C. A. Griffith M. Griffith O. L. Walker J. Kiwala S. (2016). Cancer immunogenomics: computational neoantigen identification and vaccine design. *Cold Spring Harb. Symp. Quant. Biol.* 81 105–111. 10.1101/sqb.2016.81.030726 28389595 PMC5702270

[B58] Hussein H. A. Borrel A. Geneix C. Petitjean M. Regad L. Camproux A. C. (2015). PockDrug-Server: a new web server for predicting pocket druggability on holo and apo proteins. *Nucleic Acids Res.* 43 W436–W442. 10.1093/nar/gkv462 25956651 PMC4489252

[B59] Iqbal N. Iqbal N. (2014). Human epidermal growth factor receptor 2 (HER2) in cancers: overexpression and therapeutic implications. *Mol. Biol. Int.* 2014:852748. 10.1155/2014/852748 25276427 PMC4170925

[B60] Janeway C. A. Jr. Travers P. Walport M. (2001). *Immunobiology: The Immune System in Health and Disease*, 5th Edn, (New York, NY: Garland Science). <>

[B61] Jaskolski M. Dauter Z. Wlodawer A. (2014). A brief history of macromolecular crystallography, illustrated by a family tree and its Nobel fruits. *FEBS J.* 281 3985–4009. 10.1111/febs.12796 24698025 PMC6309182

[B62] Jawad M. Seedhouse C. Mony U. Grundy M. Russell N. H. Pallis M. (2010). Analysis of factors that affect in vitro chemosensitivity of leukaemic stem and progenitor cells to gemtuzumab ozogamicin (Mylotarg) in acute myeloid leukaemia. *Leukemia* 24 74–80. 10.1038/leu.2009.199 19776761

[B63] Jiang J. Zheng C. Zhu K. Liu J. Sun N. Wang C. (2015). Quantum chemistry calculation-aided structural optimization of combretastatin A-4-like tubulin polymerization inhibitors: improved stability and biological activity. *J. Med. Chem.* 58 2538–2546. 10.1021/acs.jmedchem.5b00118 25689111

[B64] Kaufman M. Urbain J. Thomas R. (1985). Towards a logical analysis of the immune response. *J. Theor. Biol.* 114 527–561. 10.1016/S0022-5193(85)80042-4 3875000

[B65] Kelderhouse L. E. Chelvam V. Wayua C. Mahalingam S. Poh S. Kularatne S. A. (2013). Development of tumor-targeted near infrared probes for fluorescence guided surgery. *Bioconjug. Chem.* 24 1075–1080. 10.1021/bc400131a 23642154

[B66] Kim J. H. Resende R. Wennekes T. Chen H. M. Bance N. Buchini S. (2013). Mechanism-based covalent neuraminidase inhibitors with broad-spectrum influenza antiviral activity. *Science* 340 71–75. 10.1126/science.1232552 23429702

[B67] Kim M. T. Chen Y. Marhoul J. Jacobson F. (2014). Statistical modeling of the drug load distribution on trastuzumab emtansine (Kadcyla), a lysine-linked antibody drug conjugate. *Bioconjug. Chem.* 25 1223–1232. 10.1021/bc5000109 24873191

[B68] Kong X. Chen L. Jiao L. Jiang X. Lian F. Lu J. (2014). Astemizole arrests the proliferation of cancer cells by disrupting the EZH2-EED interaction of polycomb repressive complex 2. *J. Med. Chem.* 57 9512–9521. 10.1021/jm501230c 25369470

[B69] Kratochwil C. Giesel F. L. Stefanova M. Benesova M. Bronzel M. Afshar-Oromieh A. (2016). PSMA-targeted radionuclide therapy of metastatic castration-resistant prostate cancer with 177Lu-Labeled PSMA-617. *J. Nucl. Med.* 57 1170–1176. 10.2967/jnumed.115.171397 26985056

[B70] Kung Sutherland M. S. Walter R. B. Jeffrey S. C. Burke P. J. Yu C. Kostner H. (2013). SGN-CD33A: a novel CD33-targeting antibody-drug conjugate using a pyrrolobenzodiazepine dimer is active in models of drug-resistant AML. *Blood* 122 1455–1463. 10.1182/blood-2013-03-491506 23770776

[B71] Kurzrock R. Gabrail N. Chandhasin C. Moulder S. Smith C. Brenner A. (2012). Safety, pharmacokinetics, and activity of GRN1005, a novel conjugate of angiopep-2, a peptide facilitating brain penetration, and paclitaxel, in patients with advanced solid tumors. *Mol. Cancer Ther.* 11 308–316. 10.1158/1535-7163.mct-11-0566 22203732

[B72] Lamba V. Ghosh I. (2012). New directions in targeting protein kinases: focusing upon true allosteric and bivalent inhibitors. *Curr. Pharm. Des.* 18 2936–2945. 10.2174/138161212800672813 22571662

[B73] Lancet J. E. (2013). New agents: great expectations not realized. *Best Pract. Res. Clin. Haematol.* 26 269–274. 10.1016/j.beha.2013.10.007 24309529

[B74] Langmead B. Salzberg S. L. (2012). Fast gapped-read alignment with Bowtie 2. *Nat. Methods* 9 357–359. 10.1038/nmeth.1923 22388286 PMC3322381

[B75] Lauschke V. M. Ingelman-Sundberg M. (2016). Precision medicine and rare genetic variants. *Trends Pharmacol. Sci.* 37 85–86. 10.1016/j.tips.2015.10.006 26705087

[B76] Lavogina D. Enkvist E. Uri A. (2010). Bisubstrate inhibitors of protein kinases: from principle to practical applications. *ChemMedChem* 5 23–34. 10.1002/cmdc.200900252 19774589

[B77] Leamon C. P. Vlahov I. R. Reddy J. A. Vetzel M. Santhapuram H. K. You F. (2014). Folate-vinca alkaloid conjugates for cancer therapy: a structure-activity relationship. *Bioconjug. Chem.* 25 560–568. 10.1021/bc400441s 24564229

[B78] LeCun Y. Bengio Y. Hinton G. (2015). Deep learning. *Nature* 521 436–444. 10.1038/nature14539 26017442

[B79] Lee J. Y. K. Cho S. S. Zeh R. Pierce J. T. Martinez-Lage M. Adappa N. D. (2017). Folate receptor overexpression can be visualized in real time during pituitary adenoma endoscopic transsphenoidal surgery with near-infrared imaging. *J. Neurosurg.* 10.3171/2017.2.jns163191 [Epub ahead of print]. 28841122 PMC10980838

[B80] Li H. (2014). Toward better understanding of artifacts in variant calling from high-coverage samples. *Bioinformatics* 30 2843–2851. 10.1093/bioinformatics/btu356 24974202 PMC4271055

[B81] Li H. Durbin R. (2009). Fast and accurate short read alignment with Burrows-Wheeler transform. *Bioinformatics* 25 1754–1760. 10.1093/bioinformatics/btp324 19451168 PMC2705234

[B82] Liu X. Ouyang S. Yu B. Liu Y. Huang K. Gong J. (2010). PharmMapper server: a web server for potential drug target identification using pharmacophore mapping approach. *Nucleic Acids Res.* 38 W609–W614. 10.1093/nar/gkq300 20430828 PMC2896160

[B83] Liu X. Shi D. Zhou S. Liu H. Liu H. Yao X. (2018). Molecular dynamics simulations and novel drug discovery. *Expert Opin. Drug Discov.* 13 23–37. 10.1080/17460441.2018.1403419 29139324

[B84] Loeb L. A. Loeb K. R. Anderson J. P. (2003). Multiple mutations and cancer. *Proc. Natl. Acad. Sci. U.S.A.* 100 776–781. 10.1073/pnas.0334858100 12552134 PMC298677

[B85] Lu J. Zeng H. Liang Z. Chen L. Zhang L. Zhang H. (2015). Network modelling reveals the mechanism underlying colitis-associated colon cancer and identifies novel combinatorial anti-cancer targets. *Sci. Rep.* 5:14739. 10.1038/srep14739 26446703 PMC4597205

[B86] Lu W. Zhang R. Jiang H. Zhang H. Luo C. (2018). Computer-aided drug design in epigenetics. *Front. Chem.* 6:57 10.3389/fchem.2018.00057 PMC5857607 29594101

[B87] Lu Y. C. Robbins P. F. (2016). Cancer immunotherapy targeting neoantigens. *Semin. Immunol.* 28 22–27. 10.1016/j.smim.2015.11.002 26653770 PMC4862880

[B88] Lundegaard C. Lamberth K. Harndahl M. Buus S. Lund O. Nielsen M. (2008). NetMHC-3.0: accurate web accessible predictions of human, mouse and monkey MHC class I affinities for peptides of length 8-11. *Nucleic Acids Res.* 36 W509–W512. 10.1093/nar/gkn202 18463140 PMC2447772

[B89] Luyckx M. Votino R. Squifflet J. L. Baurain J. F. (2014). Profile of vintafolide (EC145) and its use in the treatment of platinum-resistant ovarian cancer. *Int. J. Womens Health* 6 351–358. 10.2147/ijwh.s39696 24729732 PMC3976235

[B90] Manglik A. Lin H. Aryal D. K. McCorvy J. D. Dengler D. Corder G. (2016). Structure-based discovery of opioid analgesics with reduced side effects. *Nature* 537 185–190. 10.1038/nature19112 27533032 PMC5161585

[B91] Mao R. Shao J. Zhu K. Zhang Y. Ding H. Zhang C. (2017). Potent, selective, and cell active protein arginine methyltransferase 5 (PRMT5) inhibitor developed by structure-based virtual screening and hit optimization. *J. Med. Chem.* 60 6289–6304. 10.1021/acs.jmedchem.7b00587 28650658

[B92] Marcoux J. Champion T. Colas O. Wagner-Rousset E. Corvaia N. Van Dorsselaer A. (2015). Native mass spectrometry and ion mobility characterization of trastuzumab emtansine, a lysine-linked antibody drug conjugate. *Protein Sci.* 24 1210–1223. 10.1002/pro.2666 25694334 PMC4534172

[B93] Martincorena I. Campbell P. J. (2015). Somatic mutation in cancer and normal cells. *Science* 349 1483–1489. 10.1126/science.aab4082 26404825

[B94] Maurer A. H. Elsinga P. Fanti S. Nguyen B. Oyen W. J. Weber W. A. (2014). Imaging the folate receptor on cancer cells with 99mTc-etarfolatide: properties, clinical use, and future potential of folate receptor imaging. *J. Nucl. Med.* 55 701–704. 10.2967/jnumed.113.133074 24732155

[B95] McKenna A. Hanna M. Banks E. Sivachenko A. Cibulskis K. Kernytsky A. (2010). The genome analysis toolkit: a MapReduce framework for analyzing next-generation DNA sequencing data. *Genome Res.* 20 1297–1303. 10.1101/gr.107524.110 20644199 PMC2928508

[B96] Mesko B. (2017). The role of artificial intelligence in precision medicine. *Expert Rev. Precis. Med. Drug Dev.* 2 239–241. 10.1080/23808993.2017.1380516

[B97] Miller R. M. Paavilainen V. O. Krishnan S. Serafimova I. M. Taunton J. (2013). Electrophilic fragment-based design of reversible covalent kinase inhibitors. *J. Am. Chem. Soc.* 135 5298–5301. 10.1021/ja401221b 23540679 PMC3665406

[B98] Mohamed H. E. Mohamed A. A. Al-Ghobashy M. A. Fathalla F. A. Abbas S. S. (2018). Stability assessment of antibody-drug conjugate Trastuzumab emtansine in comparison to parent monoclonal antibody using orthogonal testing protocol. *J. Pharm. Biomed. Anal.* 150 268–277. 10.1016/j.jpba.2017.12.022 29258046

[B99] Mollica L. Decherchi S. Zia S. R. Gaspari R. Cavalli A. Rocchia W. (2015). Kinetics of protein-ligand unbinding via smoothed potential molecular dynamics simulations. *Sci. Rep.* 5:11539. 10.1038/srep11539 26103621 PMC4477625

[B100] Morris R. T. Joyrich R. N. Naumann R. W. Shah N. P. Maurer A. H. Strauss H. W. (2014). Phase II study of treatment of advanced ovarian cancer with folate-receptor-targeted therapeutic (vintafolide) and companion SPECT-based imaging agent (99mTc-etarfolatide). *Ann. Oncol.* 25 852–858. 10.1093/annonc/mdu024 24667717

[B101] Muro S. (2012). Challenges in design and characterization of ligand-targeted drug delivery systems. *J. Control. Release* 164 125–137. 10.1016/j.jconrel.2012.05.052 22709588 PMC3481020

[B102] Nara H. Sato K. Naito T. Mototani H. Oki H. Yamamoto Y. (2014a). Discovery of novel, highly potent, and selective quinazoline-2-carboxamide-based matrix metalloproteinase (MMP)-13 inhibitors without a zinc binding group using a structure-based design approach. *J. Med. Chem.* 57 8886–8902. 10.1021/jm500981k 25264600

[B103] Nara H. Sato K. Naito T. Mototani H. Oki H. Yamamoto Y. (2014b). Thieno[2,3-d]pyrimidine-2-carboxamides bearing a carboxybenzene group at 5-position: highly potent, selective, and orally available MMP-13 inhibitors interacting with the S1” binding site. *Bioorg. Med. Chem.* 22 5487–5505. 10.1016/j.bmc.2014.07.025 25192810

[B104] Nielsen M. Lundegaard C. Blicher T. Lamberth K. Harndahl M. Justesen S. (2007). NetMHCpan, a method for quantitative predictions of peptide binding to any HLA-A and -B locus protein of known sequence. *PLoS One* 2:e796. 10.1371/journal.pone.0000796 17726526 PMC1949492

[B105] Nielsen M. Lundegaard C. Worning P. Lauemoller S. L. Lamberth K. Buus S. (2003). Reliable prediction of T-cell epitopes using neural networks with novel sequence representations. *Protein Sci.* 12 1007–1017. 10.1110/ps.0239403 12717023 PMC2323871

[B106] Ota Y. Itoh Y. Kaise A. Ohta K. Endo Y. Masuda M. (2016). Targeting cancer with PCPA-Drug conjugates: LSD1 inhibition-triggered release of 4-hydroxytamoxifen. *Angew. Chem. Int. Ed. Engl.* 55 16115–16118. 10.1002/anie.201608711 27882656

[B107] Ott P. A. Hu Z. Keskin D. B. Shukla S. A. Sun J. Bozym D. J. (2017). An immunogenic personal neoantigen vaccine for patients with melanoma. *Nature* 547 217–221. 10.1038/nature22991 28678778 PMC5577644

[B108] Pappalardo F. Fichera E. Paparone N. Lombardo A. Pennisi M. Russo G. (2016). A computational model to predict the immune system activation by citrus-derived vaccine adjuvants. *Bioinformatics* 32 2672–2680. 10.1093/bioinformatics/btw293 27162187

[B109] Pappalardo F. Pennisi M. Ricupito A. Topputo F. Bellone M. (2014). Induction of T-cell memory by a dendritic cell vaccine: a computational model. *Bioinformatics* 30 1884–1891. 10.1093/bioinformatics/btu059 24603984

[B110] Pappalardo F. Russo G. Tshinanu F. M. Viceconti M. (2018). In silico clinical trials: concepts and early adoptions. *Brief. Bioinform.* 10.1093/bib/bby043 [Epub ahead of print]. 29868882

[B111] Parigger J. Zwaan C. M. Reinhardt D. Kaspers G. J. (2016). Dose-related efficacy and toxicity of gemtuzumab ozogamicin in pediatric acute myeloid leukemia. *Expert Rev. Anticancer Ther.* 16 137–146. 10.1586/14737140.2016.1129903 26646091

[B112] Peng J. Lu J. Shen Q. Zheng M. Luo X. Zhu W. (2014). In silico site of metabolism prediction for human UGT-catalyzed reactions. *Bioinformatics* 30 398–405. 10.1093/bioinformatics/btt681 24273240

[B113] Pennisi M. Russo G. Di Salvatore V. Candido S. Libra M. Pappalardo F. (2016). Computational modeling in melanoma for novel drug discovery. *Expert Opin. Drug Discov.* 11 609–621. 10.1080/17460441.2016.1174688 27046143

[B114] Perez E. A. Barrios C. Eiermann W. Toi M. Im Y. H. Conte P. (2017). Trastuzumab emtansine with or without pertuzumab versus trastuzumab plus taxane for human epidermal growth factor receptor 2-positive, advanced breast cancer: primary results from the phase III MARIANNE study. *J. Clin. Oncol.* 35 141–148. 10.1200/jco.2016.67.4887 28056202 PMC5455677

[B115] Poot A. J. van Ameijde J. Slijper M. van den Berg A. Hilhorst R. Ruijtenbeek R. (2009). Development of selective bisubstrate-based inhibitors against protein kinase C (PKC) isozymes by using dynamic peptide microarrays. *Chembiochem* 10 2042–2051. 10.1002/cbic.200900199 19618415

[B116] Poplin R. Newburger D. Dijamco J. Nguyen N. Loy D. Gross S. S. (2017). Creating a universal SNP and small indel variant caller with deep neural networks. *bioRxiv* [Preprint]. 10.1101/092890 30247488

[B117] Predina J. D. Newton A. Deshpande C. Low P. Singhal S. (2018). Utilization of targeted near-infrared molecular imaging to improve pulmonary metastasectomy of osteosarcomas. *J. Biomed. Opt.* 23 1–4. 10.1117/1.jbo.23.1.016005 29302953 PMC5753425

[B118] Predina J. D. Newton A. Connolly C. Singhal S. (2017a). Folate receptor-targeted molecular imaging improves identification of malignancy during pulmonary resection: a case report. *J. Cardiothorac. Surg.* 12:110. 10.1186/s13019-017-0664-7 29202877 PMC5716373

[B119] Predina J. D. Newton A. D. Keating J. Barbosa E. M. Jr. Okusanya O. (2017b). Intraoperative molecular imaging combined with positron emission tomography improves surgical management of peripheral malignant pulmonary nodules. *Ann. Surg.* 266 479–488. 10.1097/sla.0000000000002382 28746152 PMC11073793

[B120] Ragoza M. Hochuli J. Idrobo E. Sunseri J. Koes D. R. (2017). Protein-ligand scoring with convolutional neural networks. *J. Chem. Inf. Model.* 57 942–957. 10.1021/acs.jcim.6b00740 28368587 PMC5479431

[B121] Regoes R. R. Barber D. L. Ahmed R. Antia R. (2007). Estimation of the rate of killing by cytotoxic T lymphocytes in vivo. *Proc. Natl. Acad. Sci. U.S.A.* 104 1599–1603. 10.1073/pnas.0508830104 17242364 PMC1785271

[B122] Rodrik-Outmezguine V. S. Okaniwa M. Yao Z. Novotny C. J. McWhirter C. Banaji A. (2016). Overcoming mTOR resistance mutations with a new-generation mTOR inhibitor. *Nature* 534 272–276. 10.1038/nature17963 27279227 PMC4902179

[B123] Schumacher T. N. Schreiber S. R. (2015). Neoantigens in cancer immunotherapy. *Science* 348 69–74. 10.1126/science.aaa4971 25838375

[B124] Sendur M. A. Aksoy S. Altundag K. (2013). Cardiotoxicity of novel HER2-targeted therapies. *Curr. Med. Res. Opin.* 29 1015–1024. 10.1185/03007995.2013.807232 23692263

[B125] Serafimova I. M. Pufall M. A. Krishnan S. Duda K. Cohen M. S. Maglathlin R. L. (2012). Reversible targeting of noncatalytic cysteines with chemically tuned electrophiles. *Nat. Chem. Biol.* 8 471–476. 10.1038/nchembio.925 22466421 PMC3657615

[B126] Singh R. Erickson H. K. (2009). Antibody-cytotoxic agent conjugates: preparation and characterization. *Methods Mol. Biol.* 525 445–467. 10.1007/978-1-59745-554-1_23 19252846

[B127] Siramshetty V. B. Eckert O. A. Gohlke B. O. Goede A. Chen Q. Devarakonda P. (2018). SuperDRUG2: a one stop resource for approved/marketed drugs. *Nucleic Acids Res.* 46 D1137–D1143. 10.1093/nar/gkx1088 29140469 PMC5753395

[B128] Śledź P. Caflisch A. (2018). Protein structure-based drug design: from docking to molecular dynamics. *Curr. Opin. Struct. Biol.* 48 93–102. 10.1016/j.sbi.2017.10.010 29149726

[B129] Srinivasarao M. Low P. S. (2017). Ligand-targeted drug delivery. *Chem. Rev.* 117 12133–12164. 10.1021/acs.chemrev.7b00013 28898067

[B130] Stein E. M. Walter R. B. Erba H. P. Fathi A. T. Advani A. S. Lancet J. E. (2017). A phase 1 trial of vadastuximab talirine as monotherapy in patients with CD33 positive acute myeloid leukemia (AML). *Blood* 131 387–396. 10.1182/blood-2017-06-789800 29196412 PMC5813721

[B131] Szegedy C. Vanhoucke V. Ioffe S. Shlens J. Wojna Z. R. (2015). “Rethinking the inception architecture for computer vision,” in *Proceedings of the IEEE Conference on Computer Vision and Pattern Recognition (CVPR)*, Las Vegas, NV.

[B132] Tao C. Sun J. Zheng W. J. Chen J. Xu H. (2015). Colorectal cancer drug target prediction using ontology-based inference and network analysis. *Database* 2015:bav015. 10.1093/database/bav015 25818893 PMC4375358

[B133] Thomas S. E. Mendes V. Kim S. Y. Malhotra S. Ochoa-Montano B. Blaszczyk M. (2017). Structural biology and the design of new therapeutics: from HIV and cancer to mycobacterial infections: a paper dedicated to John Kendrew. *J. Mol. Biol.* 429 2677–2693. 10.1016/j.jmb.2017.06.014 28648615

[B134] Valant C. Robert Lane J. Sexton P. M. Christopoulos A. (2012). The best of both worlds Bitopic orthosteric/allosteric ligands of g protein-coupled receptors. *Annu. Rev. Pharmacol. Toxicol.* 52 153–178. 10.1146/annurev-pharmtox-010611-134514 21910627

[B135] van Dam G. M. Themelis G. Crane L. M. Harlaar N. J. Pleijhuis R. G. Kelder W. (2011). Intraoperative tumor-specific fluorescence imaging in ovarian cancer by folate receptor-alpha targeting: first in-human results. *Nat. Med.* 17 1315–1319. 10.1038/nm.2472 21926976

[B136] van de Donk N. W. Dhimolea E. (2012). Brentuximab vedotin. *MAbs* 4 458–465. 10.4161/mabs.20230 22684302 PMC3499340

[B137] van Wandelen L. T. van Ameijde J. Ismail-Ali A. F. van Ufford H. C. Vijftigschild L. A. Beekman J. M. (2013). Cell-penetrating bisubstrate-based protein kinase C inhibitors. *ACS Chem. Biol.* 8 1479–1487. 10.1021/cb300709g 23621550

[B138] van Wandelen L. T. van Ameijde J. Mady A. S. Wammes A. E. Bode A. Poot A. J. (2012). Directed modulation of protein kinase C isozyme selectivity with bisubstrate-based inhibitors. *ChemMedChem* 7 2113–2121. 10.1002/cmdc.201200349 23139239

[B139] Vergote I. Leamon C. P. (2015). Vintafolide: a novel targeted therapy for the treatment of folate receptor expressing tumors. *Ther. Adv. Med. Oncol.* 7 206–218. 10.1177/1758834015584763 26136852 PMC4480526

[B140] Vergote I. B. Marth C. Coleman R. L. (2015). Role of the folate receptor in ovarian cancer treatment: evidence, mechanism, and clinical implications. *Cancer Metastasis Rev.* 34 41–52. 10.1007/s10555-014-9539-8 25564455

[B141] Villamor N. Montserrat E. Colomer D. (2003). Mechanism of action and resistance to monoclonal antibody therapy. *Semin. Oncol.* 30 424–433. 10.1016/S0093-7754(03)00261-6 12939711

[B142] Vlahov I. R. Leamon C. P. (2012). Engineering folate-drug conjugates to target cancer: from chemistry to clinic. *Bioconjug. Chem.* 23 1357–1369. 10.1021/bc2005522 22667324

[B143] Welslau M. Dieras V. Sohn J. H. Hurvitz S. A. Lalla D. Fang L. (2014). Patient-reported outcomes from EMILIA, a randomized phase 3 study of trastuzumab emtansine (T-DM1) versus capecitabine and lapatinib in human epidermal growth factor receptor 2-positive locally advanced or metastatic breast cancer. *Cancer* 120 642–651. 10.1002/cncr.28465 24222194

[B144] Wu R. Wang S. Zhou N. Cao Z. Zhang Y. (2010). A proton-shuttle reaction mechanism for histone deacetylase 8 and the catalytic role of metal ions. *J. Am. Chem. Soc.* 132 9471–9479. 10.1021/ja103932d 20568751 PMC2908479

[B145] Xia W. Hilgenbrink A. R. Matteson E. L. Lockwood M. B. Cheng J. X. Low P. S. (2009). A functional folate receptor is induced during macrophage activation and can be used to target drugs to activated macrophages. *Blood* 113 438–446. 10.1182/blood-2008-04-150789 18952896

[B146] Zhang P. Brusic V. (2014). Mathematical modeling for novel cancer drug discovery and development. *Expert Opin. Drug Discov.* 9 1133–1150. 10.1517/17460441.2014.941351 25062617

[B147] Zhao H. Dong J. Lafleur K. Nevado C. Caflisch A. (2012). Discovery of a novel chemotype of tyrosine kinase inhibitors by fragment-based docking and molecular dynamics. *ACS Med. Chem. Lett.* 3 834–838. 10.1021/ml3001984 24900387 PMC4025670

[B148] Zhou J. Li M. Chen N. Wang S. Luo H. B. Zhang Y. (2015). Computational design of a time-dependent histone deacetylase 2 selective inhibitor. *ACS Chem. Biol.* 10 687–692. 10.1021/cb500767c 25546141 PMC4372102

[B149] Zhou W. Ercan D. Chen L. Yun C. H. Li D. Capelletti M. (2009). Novel mutant-selective EGFR kinase inhibitors against EGFR T790M. *Nature* 462 1070–1074. 10.1038/nature08622 20033049 PMC2879581

[B150] Zhou W. Hur W. McDermott U. Dutt A. Xian W. Ficarro S. B. (2010). A structure-guided approach to creating covalent FGFR inhibitors. *Chem. Biol.* 17 285–295. 10.1016/j.chembiol.2010.02.007 20338520 PMC2920453

[B151] Zhu F. Li X. X. Yang S. Y. Chen Y. Z. (2018). Clinical success of drug targets prospectively predicted by in silico study. *Trends Pharmacol. Sci.* 39 229–231. 10.1016/j.tips.2017.12.002 29295742

[B152] Zuboy J. (2000). Food and Drug Administration advisory committee supports approval of antibody agent for the treatment of older Americans with acute myeloid leukemia. *Curr. Treat. Options Oncol.* 1:93. 12061372

